# Nanotechnology strategies for enhancing productivity, sulphur use efficiency and soil fertility in groundnut-mustard cropping systems

**DOI:** 10.1038/s41598-025-33174-5

**Published:** 2026-01-06

**Authors:** Suwa Lal Yadav, K. C. Patel, Dileep Kumar, Dinesh Jinger, Lalu Prasad Yadav, Devilal Birla, Manish Yadav, Rajendra Kumar Yadav, Indra Raj Yadav, Lakshman, Tatiana Minkina, Vishnu D. Rajput, Pooja Yadav, Vijay Singh Meena

**Affiliations:** 1 Department of Soil Science and Agricultural Chemistry, Anand Agricultural University, Anand, Gujarat India; 2 Micronutrients Research Centre, Anand Agricultural University, Anand, Gujarat India; 3 Research Centre, ICAR-Indian Institute of Soil and Water Conservation, Vasad, Gujarat India; 4 Central Horticultural Experiment Station (ICAR-CIAH), Godhra-Vadodara Highway, Vejalpur, Gujarat India; 5 Department of Agronomy, Anand Agricultural University, Anand, Gujarat India; 6 Department of Soil Science, Punjab Agricultural University, Ludhiana, Punjab India; 7 Agriculture University, Kota, Rajasthan India; 8 Department of Soil Science, Rajmata Vijayaraje Scindia Krishi Vishwa Vidyalaya, Gwalior, Madhya Pradesh India; 9 Department of Agronomy, Chandra Shekhar Azad University of Agriculture and Technology, Kanpur, Uttar Pradesh India; 10 Academy of Biology and Biotechnology, Southern Federal University, Rostov-on-Don, Russia; 11 Raffles University, Neemrana, Rajasthan India; 12 Maharana Pratap University of Agriculture and Technology, Udaipur, Rajasthan India; 13 ICAR-Indian Agricultural Research Institute, Regional Station, Pusa Samastipur, Bihar India

**Keywords:** Micronutrients, Sulphur use efficiency, Food security, Environmental sciences, Environmental impact

## Abstract

This study aimed to evaluate the impact of sulfur application, particularly sulfur nanoparticles (SNPs), on yield, nutrient content, uptake, and soil sulfur dynamics in a groundnut-mustard cropping system. Two year (2021–2022) study was conducted at Anand Agricultural University, Gujarat, India. Eleven treatments of SNPs and elemental sulfur were applied via fertigation, and their effects on groundnut-mustard cropping system performance and soil properties were analyzed. The highest chlorophyll content, seed, haulm, and stover yield, as well as increased sulfur, iron, zinc, manganese, and copper content in plant parts, were observed under recommended dose of fertilizer (RDF) + SNPs at 3.0 mg S/kg soil (split application: half at sowing, half at 1 month after sowing). Sulfur fractions and Diethylene Triamine Penta Acetic acid extractable micronutrients were highest at RDF + SNPs at 4.0 mg S/kg soil, split similarly. The lowest values were recorded under control treatment. The optimized application of SNPs significantly improved yield, nutrient uptake, and sulfur use efficiency in the groundnut-mustard system, offering a sustainable alternative to conventional sulfur fertilizers. RDF + SNPs at 3.0 mg S/kg soil in a split dose is recommended as an optimum sulfur management strategy under controlled conditions. This approach supports sustainable agricultural practices and efficient nutrient management, ensuring long-term soil health and productivity.

**Supplementary Information:**

The online version contains supplementary material available at 10.1038/s41598-025-33174-5.

## Introduction

Farmers have been using macronutrients to fetch higher production as well as returns from agricultural land since the commencement of the green revolution. On the other hand, in developing countries, the cost of fertilizer can be significantly very high and it is often the limiting factor for food security, quality and supply and it had very low use efficiency^1–3^. Therefore, the application of secondary nutrients is the need of the hour. Among the secondary nutrients sulphur (S) is the most important nutrient because it is not applied in the required amount by the growers and it plays vital role in oilseed crops^4^. Sulphur is a constituent of amino acids, vitamins, and enzymes, and plays a vital role in photosynthesis, protein synthesis, and oil production^5,6^. Its deficiency can lead to stunted growth, yellowing of leaves, which ultimately leads to reduced photosynthesis and yield. Due to itsrole in the synthesis of proteins and enzymes, which are necessary for the intake and utilisation of other macro and micronutrients, sulphur fertilisation can increase the content and uptake of other micronutrients, including zinc, iron, and manganese^6,7^. A crucial component of many plant systems’ biochemical activities, sulphur (S) is a nutrient that is necessary for plant growth^6,8^. Traditional S fertilization methods have often led to inefficiencies, such as uneven distribution, and leaching, contributing to environmental concerns^6^. Moreover, in the quest for sustainable and efficient agricultural practices, researchers and agricultural experts have turned their attention towards innovative solutions to enhance crop yields while minimizing environmental impact^9,10^. One such ground-breaking advancement is the development and application of sulphur nano-particles (SNPs), an innovative technique with the potential to completely transform contemporary agriculture^11,12^. However, the development of SNPs presents a viable way to address these issues by utilising the special qualities of nanomaterials to give S to plants more efficiently^13,14^.

Sulphur nano particles involve the manipulation of S compounds at the nanoscale, typically in the range of 1 to 100 nm^15^. This nano-structuring imparts novel properties to the S particles, enhancing their solubility, reactivity, and ability to interact with plant roots^16^. By encapsulating or attaching SNPs to carrier materials, the controlled release of S can be achieved, ensuring a more sustained and efficient nutrient supply to plants throughout their growth stages^17,18^. This targeted delivery system minimizes wastage, reduces environmental pollution, and optimizes the utilization of S resources. The benefits of SNPs extend beyond improved nutrient delivery^19,20^. These nanoparticles can also serve as platforms for the controlled release of other essential nutrients. Additionally, the nanomaterials used in these fertilizers can potentially enhance the moisture-carrying capacity of soil, contributing to soil health and reducing the need for excessive irrigation^21,22^. Therefore, the use of SNPs has the potential to support more sustainable farming methods, lower input costs, and higher crop output^23–25^.

Groundnut (*Arachis hypogea*), also known as peanut, is a nitrogen-fixing crop that comes under the Fabaceae family. It is grown for its edible seeds, which develop belowground^26,27^. Groundnut have high amount of protein, oil, and other vital nutrients, it is a significant food and oil crop in the country as well as world^27^. India is one of the top three groundnut-producing nations in the world. It ranks second next to China while, in India, Gujarat, Rajasthan, and Tamil Nadu are the major producing states during the year 2020–21. A total of 10.24 Mt of groundnuts are produced annually on 6.02 million hectares of farmed land, with an average yield of 1703 kg/ha^28^. The area under groundnut constitutes approximately 3.3 per cent of the net sown area in India. In Tamil Nadu, groundnut productivity is the highest at 2502 kg/ha; in Gujarat, it is approximately 1911 kg/ha. In Gujarat, it is grown in about 2.16 M ha with a total production of about 4.13 Mt annually^28^. India is exporting 29,873 and 8493 Mt of groundnut oil meals in 2022–23 and 2023–24 respectively^28^.

Similarly, Indian mustard (*Brassica juncea*) is also an important oilseed crop^29^. Mustard seeds vary in colour and flavour based on the species, are widely used as condiments, spices, and as a source of edible oil^30^. Mustard is not only valued for its culinary applications but also for its potential health benefits because it contains phytochemicals and antioxidants^31^. India is next after Nepal, Canada and China in production of mustard in the financial year of 2021–22. While, In India, Gujarat is next after Rajasthan, Madhya Pradesh, Uttar Pradesh, Haryana, Jharkhand and Assam in area and production^28^. With an average yield of 1703 kg/ha and a total production of 11.96 Mt, mustard is grown on roughly 6.7 million hectares. The state of Gujarat has roughly 1976 kg/ha of mustard yield, whereas the maximum productivity is found in the state of Haryana, with 2028 kg/ha. In Gujarat, mustard is grown in an about 2.14 Mha area with a total production of about 4.24 Mt annually^28^. India is exporting 2,296,943 and 894,117 Mt of oil meals in 2022–23 and 2023–24 respectively^28^. To enhance the oil seed production as well as quality concern balanced fertilization is indispensable in these crops^32^.

Sulphur plays a critical role in the growth and productivity of oilseed crops, particularly groundnut and mustard^33,34^. It is essential for the synthesis of amino acids (cysteine and methionine), proteins, and enzymes, and directly influences oil content and quality^35,36^. Sulphur deficiency, widespread in Indian soils, often leads to reduced yields and poor oil quality in oilseeds. Mustard has a high sulphur demand due to its glucosinolate content, while groundnut requires adequate sulphur for pod development and seed protein^37,38^. Enhancing sulphur use efficiency is thus crucial for sustaining oilseed production^39^, and emerging nanotechnology-based sulphur fertilizers offer promising solutions to improve uptake, minimize losses, and boost crop performance under diverse agro-ecological conditions^40,41^. Despite the crucial role of sulphur in plant growth, its deficiency in soils due to imbalanced fertilization remains a significant challenge, particularly in sulphur-depleted regions. Traditional sulphur fertilizers often exhibit low use efficiency, leading to nutrient losses and environmental concerns. This study hypothesizes that sulphur nanoparticles (SNPs) can enhance nutrient uptake and sulphur use efficiency compared to conventional sources. Therefore, the objectives of this research are: (i) to evaluate the impact of sulphur application on the yield of groundnut and mustard, addressing the gap in optimized sulphur fertilization for improved productivity; (ii) to assess the influence of sulphur application on nutrient enrichment in the groundnut-mustard system, hypothesizing that SNPs enhance micronutrient availability and uptake; and (iii) to investigate the effect of sulphur application on sulphur dynamics and its use efficiency, filling the research gap on how nanoparticle-based sulphur fertilizers improve sulphur fractions and overall soil health.

## Materials and methods

### Investigation site and growing condition

Throughout the *rabi* (mustard) and *kharif* (groundnut) seasons of 2021–2022, the pot experiment was conducted in Polyhouse at the Centre for Advanced Research in Plant Tissue Culture, Anand Agricultural University, Anand (Gujarat). The experimental soil was loamy sand in texture, neutral in reaction with the pH (7.78), EC (0.30 dS/m)^42^, low in organic carbon 3.56 g/kg^43^, medium in available phosphorus 30.44 kg/ha^44^, and potassium 210.04 kg/ha^45^, low in available sulphur 5.16 ppm^46^, medium in Zn, Fe, Mn and Cu was 0.65, 4.46, 5.88 and 1.05 ppm respectively^47^.

### Experimental design and treatments

**Fig. 1:**
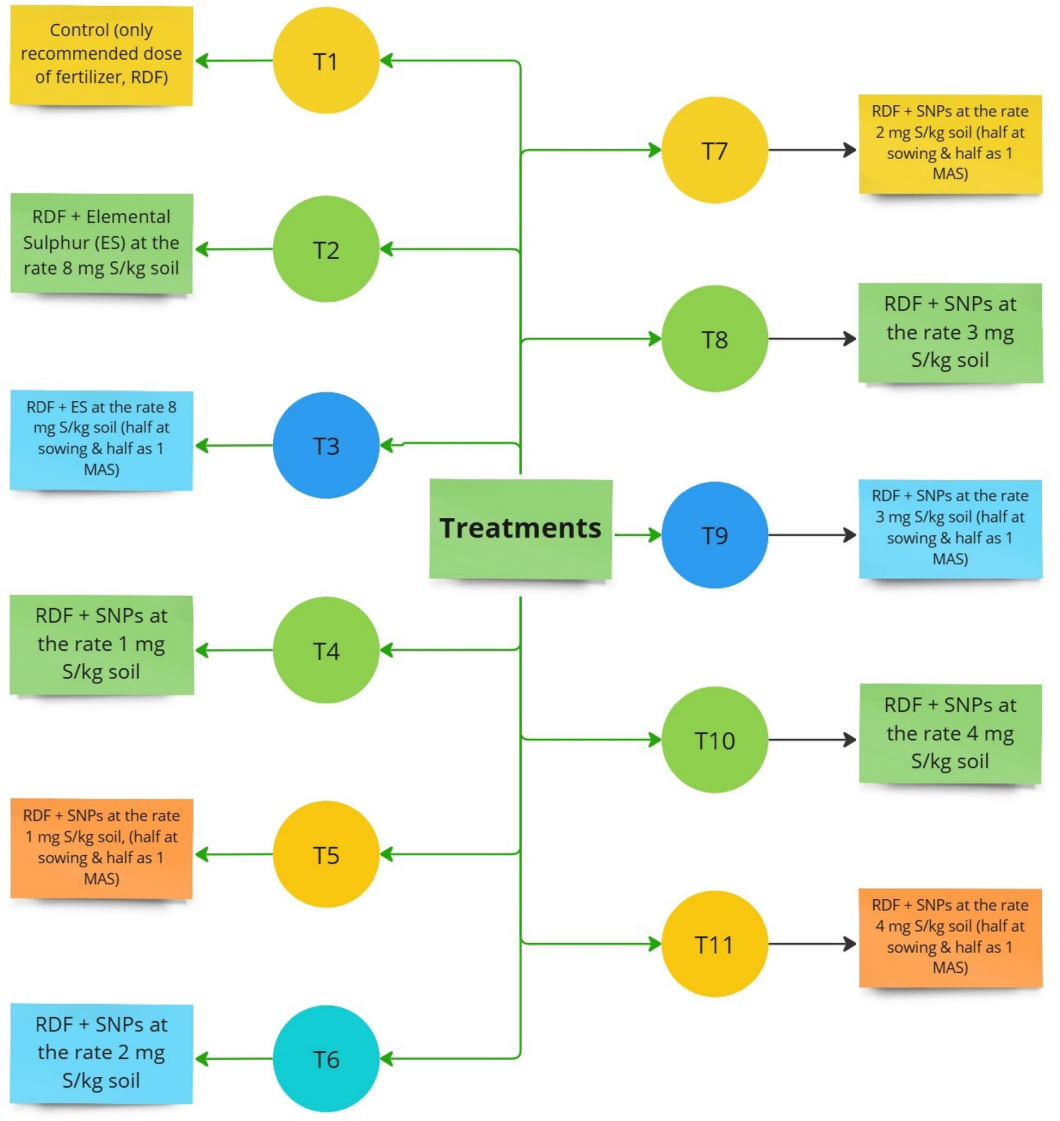
Treatment details.

The current study was carried out using a completely randomized block design with 11 treatment and four replications (Fig. 1). The treatment details of this experiment were as control (only recommended dose of fertilizer, RDF), RDF + elemental sulphur (ES) at the rate 8 mg S/kg soil, RDF + ES at the rate 8 mg S/kg soil (half at sowing and half as 1 MAS), RDF + SNPs at the rate 1 mg S/kg soil, RDF + SNPs at the rate 1 mg S/kg soil, (half at sowing and half as 1 MAS), RDF + SNPs at the rate 2 mg S/kg soil, RDF + SNPs at the rate 2 mg S/kg soil (half at sowing and half as 1 MAS), RDF + SNPs at the rate 3 mg S/kg soil, RDF + SNPs at the rate 3 mg S/kg soil (half at sowing and half as 1 MAS), RDF + SNPs at the rate 4 mg S/kg soil, RDF + SNPs at the rate 4 mg S/kg soil (half at sowing and half as 1 MAS). The RDF and sulphur treatments were applied through fertigation as per the treatments mentioned earlier and the flow rate of the dripper was kept at 4 L water per hour. The recommended dose of fertilizers (RDF) was applied using urea (46% N), muriate of potash (60% K₂O), and single super phosphate (16% P₂O₅, 11–12% S) as nutrient sources. The S treatments and RDF was applied in both the crops of *kharif* and *rabi* season as per treatment details.

### Elemental sulphur (ES) and SNPs

**Fig. 2:**
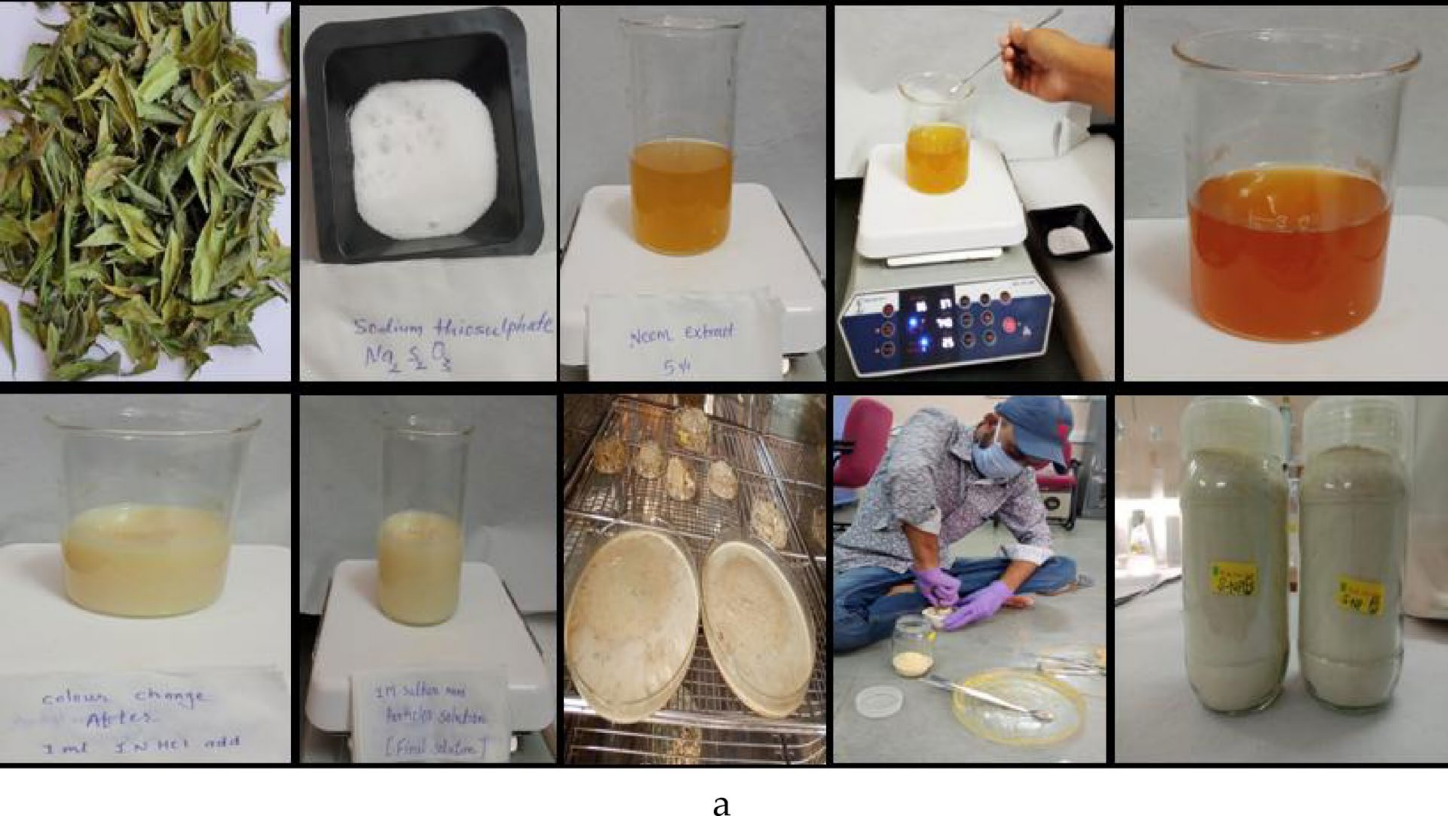

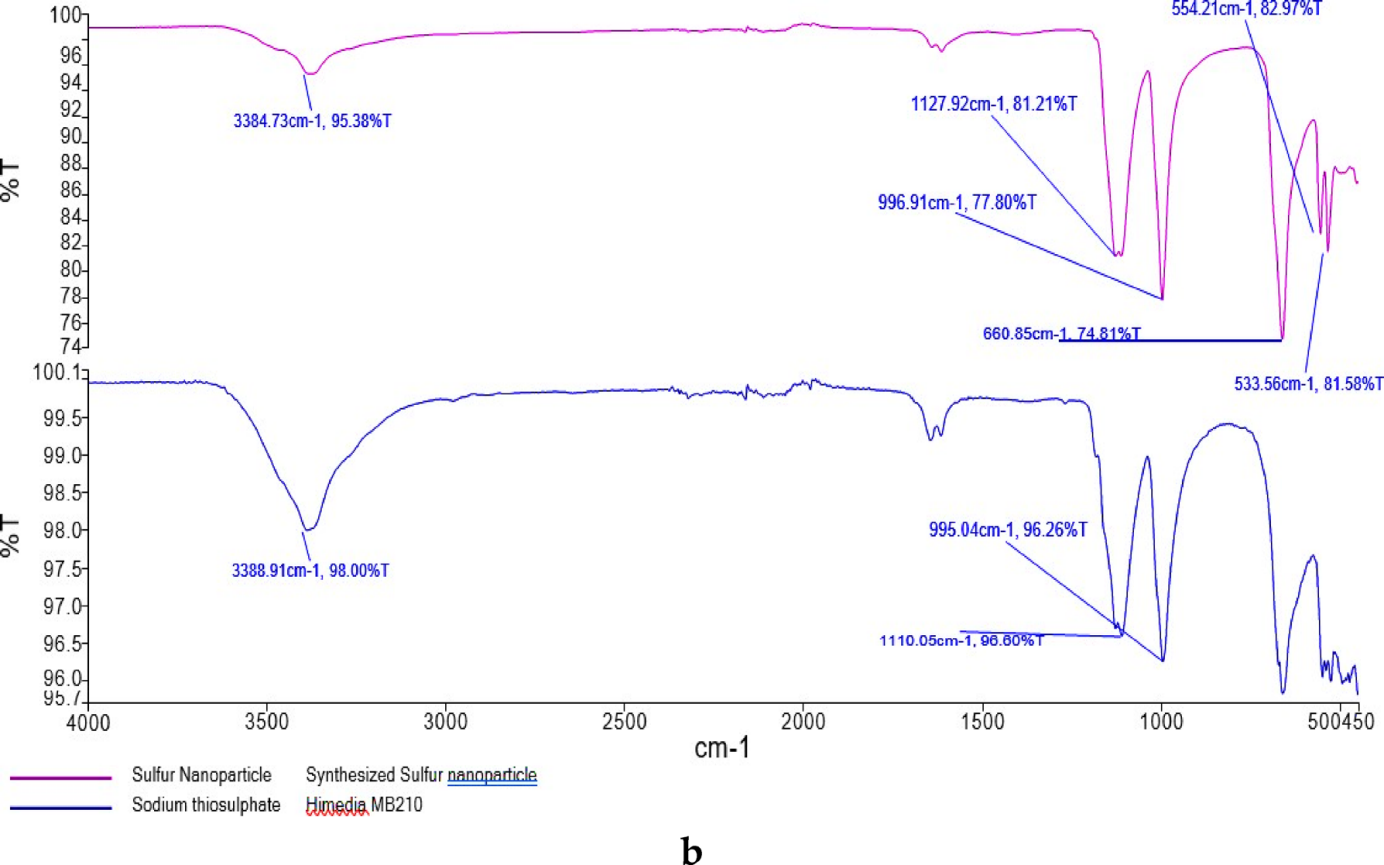
(**a**) Systematic representation of SNPs preparation using neem leaves and sodium thiosulphate (**b**) Combined FTIR spectrum of sodium thiosulphate and synthesized sulphur nanoparticle.

The ES fertilizer was brought from SML (sulphur mills limited) having 90% ES on w/w basis (Fig. 2a). The sulphur nano particle was synthesized at AAU, Anand, Gujarat, via the green synthesis method^48^ in which 5 g neem leaf powder was taken in 100 mL milli Q (Ultra-pure) water and boiled it in microwave oven for 3–5 min. The boiled extract was cooled and filtered by a sieve to get clear supernatant after that it was filtered with Whatman No 42. Volume of this extract was made up to 100 mL in a 250 mL of beaker and it was put on magnetic stirrer. After that 15.81 g of 1 M Na_2_S_4_O_7_.5H_2_O was added in it pinch by pinch in 5% neem leaf extract under constant stirring condition. After the addition of sodium thiosulphate, it was allowed to stand for 20–30 min on a magnetic stirrer and then add 1N 1–2 mL HCl till the yellow colour changed. The solution was kept on stirrer for 10–15 min and after that 1 mL of prepared solution for DLS instrument. Synthesized SNPs were kept in a refrigerator for overnight and then dried at 60 °C in hot air oven till the complete removal of water and dried powder obtained was used without any modification (Figs. 2a, b). The synthesized SNPs powder was also characterized using the UV- XRD (Fig. 1) and found that the particle has a spherical shape. The synthesized SNPs were analyzed using di-acid digestion (HNO_3_:HClO_4_, 3:1 ratio) procedure for total sulphur estimation and it had 35% sulphur on a dry weight basis.

### Crop management

Groundnut (GG-34) and mustard (GM-4) were raised under a polyhouse condition. Groundnut was sowed on 1st July 2021 and harvested on 20th October 2021. Mustard was sown on 20th November 2021 and harvested on 09th March 2022. Both the crops were sown at 2–3 cm deep manually and harvested manually. The RDF for groundnut and mustard was 25–50-00 and 50–25-00 kg/ha of N, P and K, respectively. Seeds were treated with Agrosan GN fungicide not with rhizobium culture. Two hand weeding were done at 25 and 45 DAS, and drip irrigation was applied frequently with flow rate of 4 L/hour. Standard crop practices were followed, and dimethoate was sprayed twice on mustard for aphid control.

### Content and uptake

Sulphur content was determined using the standard procedure^49^. The plant samples seed, haulm and stover were washed, dried and it was digested in di-acid mixture of HNO_3_: HClO_4_ till a white colour appeared. The digested solution was diluted to 50 mL and analyzed using spectrophotometer at 430 nm wavelength. The sulphur upta was calculated using given formula^50^.

1 $$Sulphur upke\left( {mg/pot} \right) = \frac{{Sulphur conent\left( \% \right) \times Dry matter yield\left( {g/pot} \right)}}{100}$$

Micronutrient uptake was calculated as per the given formulas^50^.

2 $$Micronutrient uptake\left( {mg/pot} \right) = \frac{{Nutrient content\left( {mg/kg} \right) \times Dry matter yield\left( {g/pot} \right)}}{1000}$$

3 $$SUE\left( \% \right) = \frac{{\begin{array}{*{20}c} {Sulphur uptakefromfertilized plot \left( {mg/pot} \right) - Sulphur uptake from} \\ {unfertilized plot\left( {mg/pot} \right)} \\ \end{array} }}{{Sulphur applied \left( {mg/pot} \right)}}$$

### Sulphur fractions in soil

Sulphur dynamics (fractions) were determined using a destructive method for each fraction. The CaCl_2_ (0.15%) method was used for the determination of water soluble, heat soluble and adsorbed sulphate sulphur^36^. Organic and total sulphur fractions in the soil were determined using standard procedure^51,52^. Non-sulphate sulphur was determined by computing the difference between total sulphur and the sum of organic and sulphate sulphur^46^.

### Data analysis and interpretation

The analyses of variance (ANOVA) of the parameters were performed using the F-test^53^. The treatment means comparisons were carried out by Duncan’s New Multiple Range test (DNMRT) using SPSS 16.0 for Windows (SPSS Inc., Chicago, IL, USA). The heat map of correlation was generated using Python software.

## Results

### Chlorophyll content of groundnut and mustard

Treatments, RDF + SNPs at the rate 4.0 mg S/kg soil, recorded significantly higher chlorophyll content than the rest of the treatments barring RDF + sulphur nanoparticles at the rate 4.0 mg S/kg soil, (half at sowing and half as 1 MAS), at 30 DAS, respectively, while, significantly higher chlorophyll content was registered with the application of RDF + sulphur nanoparticles at the rate 3.0 mg S/kg soil, (half at sowing and half as 1 MAS) at 60 DAS over rest of the treatments. Chlorophyll content increased by 20.02 and 17.93% under RDF + sulphur nanoparticles at the rate 4.0 mg S/kg soil and RDF + sulphur nanoparticles at the rate 3.0 mg S/kg soil, (half at sowing and half as 1 MAS), respectively over control in groundnut wherever, 18.76 and 17.0% higher was obtained under mustard.

### Productivity of groundnut and mustard

Significantly remarkably pod yield /plant achieved with the application of RDF + sulphur nanoparticles at the rate 3.0 mg S/kg soil, with (half at sowing and half as 1 MAS) than rest of the treatments (Table 1). This novel approach resulted in pod yield measuring 12.50 g /plant, surpassing all other treatments. Meanwhile, lowest (11.04 g) pod yield reported at control. Increases in pod yield with the use of RDF + sulphur nanoparticles at the rate 3.0 mg S/kg soil; (half at sowing and half as 1 MAS) was in the tune of 13.22% higher over control. Notably, treatment RDF + sulphur nanoparticles at the rate 3.0 mg S/kg soil, (half at sowing and half as 1 MAS), exhibited significantly higher haulm yield/plant, which was 20.28 g than rest of the treatments barring RDF + sulphur nanoparticles at the rate 4.0 mg S/kg soil and RDF + sulphur nanoparticles at the rate 4.0 mg S/kg soil, (half at sowing and half as 1 MAS) (Table 1). The control treatment, without sulphur application recorded the lowest haulm yield. The increment in haulm yield with the fertilization of RDF + sulphur nanoparticles at the rate 3.0 mg S/kg soil, (half at sowing and half as 1 MAS) was 14.50% higher than the untreated pot.

**Table 1:** Effect of SNPs and elemental sulphur on chlorophyll concentration, yield attributes and yield of groundnut-mustard.

Treatments	Chlorophyll concentration		Pods/plant	Yield (g /plant)	
	30 DAS	60 DAS		Pod	Haulm
**Groundnut**					
**T**_**1**_	37.96 ± 1.26^d^	31.44 ± 0.98^d^	28.06 ± 1.04^b^	11.04 ± 0.40^c^	17.71 ± 0.30^ cd^
**T**_**2**_	40.94 ± 1.05^ cd^	33.69 ± 0.86^ cd^	30.24 ± 1.01^ab^	12.33 ± 0.25^ab^	18.61 ± 0.48^bcd^
**T**_**3**_	41.08 ± 0.39^ cd^	33.8 ± 0.32^ cd^	30.45 ± 0.53^ab^	11.53 ± 0.26^abc^	18.68 ± 0.32^bcd^
**T**_**4**_	38.59 ± 0.63^ cd^	31.75 ± 0.52^ cd^	28.06 ± 0.86^b^	12.41 ± 0.19^ab^	17.55 ± 0.29^d^
**T**_**5**_	39.93 ± 0.89^ cd^	32.86 ± 0.73^ cd^	28.06 ± 0.39^b^	12.32 ± 0.32^ab^	18.16 ± 0.40^ cd^
**T**_**6**_	40.03 ± 0.61^ cd^	32.94 ± 0.50^ cd^	28.06 ± 0.73^b^	11.25 ± 0.28^c^	18.2 ± 0.28^ cd^
**T**_**7**_	40.43 ± 0.90^ cd^	33.27 ± 0.72^ cd^	29.9 ± 0.53^ab^	12.5 ± 0.10^a^	18.38 ± 0.40^ cd^
**T**_**8**_	40.82 ± 0.90^ cd^	33.59 ± 0.74^ cd^	29.9 ± 0.87^ab^	11.27 ± 0.22^c^	18.56 ± 0.41^bcd^
**T**_**9**_	41.79 ± 1.03^bc^	37.08 ± 0.74^a^	32.78 ± 0.99^a^	12.51 ± 0.26^a^	20.28 ± 0.42^a^
**T**_**10**_	45.56 ± 0.98^a^	34.39 ± 0.81^bc^	31.6 ± 1.04^a^	11.38 ± 0.30^bc^	19.03 ± 0.45^abc^
**T**_**11**_	44.18 ± 0.78^ab^	36.35 ± 0.64^ab^	31.8 ± 0.57^a^	12.37 ± 0.42^ab^	19.89 ± 0.35^ab^
**Mustard**					
**T**_**1**_	35.5 ± 1.21^d^	34.79 ± 0.69^c^	90.53 ± 3.28^c^	10.44 ± 0.17^d^	20.05 ± 0.29^c^
**T**_**2**_	38.3 ± 0.98^bcd^	37.27 ± 1.26^bc^	98.23 ± 3.36^abc^	11.74 ± 0.24^ab^	21.39 ± 0.55^bc^
**T**_**3**_	38.43 ± 0.37^bcd^	37.4 ± 0.36^bc^	98.57 ± 1.56^abc^	11.65 ± 0.21^ab^	22.03 ± 0.20^ab^
**T**_**4**_	36.1 ± 0.59^ cd^	35.13 ± 0.58^c^	91.42 ± 2.43^c^	10.71 ± 0.21^ cd^	20.17 ± 0.34^c^
**T**_**5**_	37.36 ± 0.83^ cd^	36.36 ± 0.81^c^	95.11 ± 2.10^bc^	10.73 ± 0.21^ cd^	20.87 ± 0.46^bc^
**T**_**6**_	37.46 ± 0.57^ cd^	36.45 ± 0.55^c^	94.85 ± 1.81^bc^	10.84 ± 0.28^ cd^	20.92 ± 0.32^bc^
**T**_**7**_	37.83 ± 0.84^ cd^	36.81 ± 1.49^c^	95.78 ± 2.19^bc^	11.47 ± 0.10^bc^	21.13 ± 0.75^bc^
**T**_**8**_	38.2 ± 0.84^bcd^	37.17 ± 0.82^bc^	96.72 ± 3.76^bc^	11.73 ± 0.30^ab^	21.34 ± 0.47^bc^
**T**_**9**_	39.1 ± 1.16^bc^	41.02 ± 0.82^a^	106.75 ± 2.42^a^	12.39 ± 0.10^a^	23.55 ± 0.48^a^
**T**_**10**_	42.16 ± 0.92^a^	38.04 ± 0.89^abc^	99 ± 2.32^abc^	11.91 ± 0.25^ab^	22.41 ± 0.51^ab^
**T**_**11**_	41.33 ± 0.73^ab^	40.22 ± 0.71^ab^	104.66 ± 2.19^ab^	12.02 ± 0.26^ab^	23.09 ± 0.41^a^

Significantly higher seed yield of mustard /plant was obtained with the application of RDF + sulphur nanoparticles at the rate 3.0 mg S/kg soil, (half at sowing and half as 1 MAS) than the remaining treatments barring RDF + elemental S at the rate 8.0 mg S/kg soil, RDF + elemental S at the rate 8.0 mg S/kg soil, (half at sowing and half as 1 MAS), RDF + sulphur nanoparticles at the rate 3.0 mg S/kg soil, RDF + sulphur nanoparticles at the rate 4.0 mg S/kg soil and RDF + sulphur nanoparticles at the rate 4.0 mg S/kg soil, (half at sowing and half as 1 MAS). Minimum mustard seed yield was recorded under control (Table 1). The yield was registered 18.68% higher with the application of RDF + sulphur nanoparticles at the rate 3.0 mg S/kg soil, (half at sowing and half as 1 MAS) over control treatment.

Significantly higher stover yield was obtained under RDF + sulphur nanoparticles at the rate 3.0 mg S/kg soil, (half at sowing and half as 1 MAS)_,_ which was on par with treatments RDF + Elemental S at the rate 8.0 mg S/kg soil, (half at sowing and half as 1 MAS), RDF + sulphur nanoparticles at the rate 4.0 mg S/kg soil and RDF + sulphur nanoparticles at the rate 4.0 mg S/kg soil, (half at sowing and half as 1 MAS), differed significantly from rest of the treatments. The improvement in stover yield due to the application of RDF + sulphur nanoparticles at the rate 3 mg S/kg soil, (half at sowing and half as 1 MAS) was to the tune of 17.46% over control.

### Sulphur uptake by groundnut and mustard

Application of RDF + sulphur nanoparticles at the rate 3.0 mg S/kg soil, (half at sowing and half as 1 MAS), resulted in higher sulphur content (Fig. S1) and uptake by seed and haulm of groundnut (Table 2), with a value of 113.72 and 120.42 mg /pot than rest of the treatments barring treatments RDF + elemental S at the rate 8.0 mg S/kg soil, RDF + elemental S at the rate 8.0 mg S/kg soil (half at sowing and half as 1 MAS), RDF + sulphur nanoparticles at the rate 3.0 mg S/kg soil, RDF + sulphur nanoparticles at the rate 4.0 mg S/kg soil and RDF + sulphur nanoparticles at the rate 4.0 mg S/kg soil, (half at sowing and half as 1 MAS) in case of seed, while RDF + sulphur nanoparticles at the rate 4.0 mg S/kg soil, (half at sowing and half as 1 MAS) being on par in terms of sulphur uptake by haulm. The control treatment registered significantly lower sulphur uptake of 89.85 and 94.93 mg /pot, respectively than rest of the treatments. The increase in sulphur uptake by seed and haulm of groundnut with the application of RDF + sulphur nanoparticles at the rate 3.0 mg S/kg soil, (half at sowing and half as 1 MAS) was 26.57 and 26.85% higher over control only RDF treated.

**Table 2:** Effect of SNPs and elemental sulphur on sulphur uptake and use efficiency.

Treatments	Sulphur uptake (mg /pot)				Sulphur use efficiency (%)	
	**Groundnut**		**Mustard**			
	**Seed**	**Haulm**	**Seed**	**Stover**	**Groundnut**	**Mustard**
**T**_**1**_	89.85 ± 2.86^e^	94.93 ± 4.16^d^	49.67 ± 1.82f.	57.21 ± 2.48f.	-	-
**T**_**2**_	109.13 ± 3.34^abc^	105.42 ± 4.39^bcd^	62.38 ± 1.74^bc^	66.72 ± 1.96^bcde^	19.28	18.51
**T**_**3**_	101.73 ± 2.21^bcd^	106.2 ± 2.36^bcd^	62.48 ± 1.96^bc^	68.94 ± 2.10^bcd^	24.80	20.45
**T**_**4**_	104.41 ± 1.10^abcd^	94.3 ± 2.84^d^	53.65 ± 1.65^ef^	57.26 ± 1.41f.	92.86	26.87
**T**_**5**_	103.38 ± 2.54^abcd^	99.61 ± 1.27^ cd^	53.86 ± 1.53^ef^	59.37 ± 1.52^ef^	121.37	42.29
**T**_**6**_	96.57 ± 2.24^de^	99.85 ± 1.50^ cd^	55.44 ± 1.74^def^	60.46 ± 1.64^ef^	38.79	30.05
**T**_**7**_	106.79 ± 3.47^abcd^	101.06 ± 2.98^ cd^	58.91 ± 1.50^cde^	62.34 ± 2.01^def^	76.90	47.89
**T**_**8**_	98.49 ± 3.67^cde^	103.97 ± 3.92^bcd^	60.51 ± 1.57^bcd^	64.92 ± 1.65^cdef^	39.26	41.23
**T**_**9**_	114.01 ± 2.91^a^	120.42 ± 3.12^a^	69.34 ± ^a^1.01	78.63 ± 3.06^a^	110.33	91.30
**T**_**10**_	101.81 ± 2.99^bcd^	108.83 ± 2.24^bc^	64.54 ± 2.48^abc^	70.78 ± 1.36^bc^	43.09	47.39
**T**_**11**_	111.65 ± 3.22^ab^	114.91 ± 4.57^ab^	66.06 ± 1.32^ab^	74.52 ± 3.60^ab^	69.64	56.16

At treatment RDF + sulphur nanoparticles at the rate 3.0 mg S/kg soil, (half at sowing and half as 1 MAS), resulted in the highest sulphur uptake (Table 2) by seed and stover of mustard, with values of 69.34 and 78.63 mg /pot, respectively, which being on par with RDF + sulphur nanoparticles at the rate 4.0 mg S/kg soil, (half at sowing and half as 1 MAS), differed significantly from rest of the treatments. The control treatment, which consisted of pots treated solely with RDF, displayed a significantly lower sulphur uptake of 49.67 and 57.21 mg /pot, respectively than remaining treatments except for RDF + sulphur nanoparticles at the rate 1.0 mg S/kg soil and RDF + sulphur nanoparticles at the rate 1.0 mg S/kg soil, (half at sowing and half as 1 MAS) for seed and RDF + sulphur nanoparticles at the rate 1.0 mg S/kg soil, RDF + sulphur nanoparticles at the rate 1.0 mg S/kg soil, (half at sowing and half as 1 MAS), RDF + sulphur nanoparticles at the rate 2.0 mg S/kg soil and RDF + sulphur nanoparticles at the rate 2.0 mg S/kg soil, (half at sowing and half as 1 MAS) for stover. The increase in sulphur uptake by seed and stover of mustard with application of RDF + sulphur nanoparticles at the rate 3.0 mg S/kg soil, (half at sowing and half as 1 MAS) over control was 39.60 and 37.44%, respectively.

### Micronutrient content and uptake of groundnut and mustard

#### Iron (Fe) content and uptake

At treatment combining the RDF with SNPs at the rate 3 mg S/kg soil (half at sowing and half as 1 MAS), resulted in a significantly higher seed and haulm Fe content and uptake, with a value of 50.1, 352.0 ppm, 1.25 and 14.29 mg/pot, respectively (Table 3, Figs. 4 and 5). At RDF + SNPs at the rate 3 mg S/kg soil, RDF + SNPs at the rate 4 mg S/kg soil and RDF + SNPs at the rate 4 mg S/kg soil (half at sowing and half as 1 MAS) for Fe uptake by seed and haulm. The control treatment, which consisted of pots treated solely with RDF, displayed a significantly lower seed Fe content (41.1 and 311.8 ppm) and uptake (0.91 and 11.04 mg/pot), respectively (Fig. S2). The percentage increase in Fe content and uptake of seed and haulm was 21.7, 12.8, 37.36 and 29.34% respectively under RDF + SNPs at the rate 3 mg S/kg soil (half at sowing and half as 1 MAS) over control.

**Table 3:** Effect of SNPs and ES on micronutrient content of groundnut and mustard.

Treatments	Micronutrient’s content (ppm)							
	Iron		Zinc		Manganese		Copper	
	Seed	Haulm	Seed	Haulm	Seed	Haulm	Seed	Haulm
Groundnut								
**T**_**1**_	41.17 ± 0.47^d^	311.89 ± 5.47^e^	26.06 ± 0.72^d^	16.39 ± 0.71f.	15.16 ± 0.66^e^	28.24 ± 1.52^d^	9.79 ± 0.25^d^	10.94 ± 0.63^c^
**T**_**2**_	45.37 ± 1.85^bc^	334.93 ± 7.14^abcde^	30.07 ± 0.55^abc^	20.48 ± 0.38^bc^	18.94 ± 0.35^abc^	33.84 ± 0.62^ab^	11.52 ± 0.14^ab^	13.67 ± 0.43^b^
**T**_**3**_	47.62 ± 0.33^ab^	336.71 ± 2.32^abcd^	29.37 ± 0.21^abc^	20.59 ± 0.14^bc^	19.04 ± 0.13^abc^	33.52 ± 0.38^ab^	11.6 ± 0.20^ab^	13.75 ± 0.09^b^
**T**_**4**_	43.55 ± 0.88^ cd^	314.78 ± 9.85^de^	27.84 ± 0.40^ cd^	17.94 ± 0.38^ef^	16.76 ± 0.35^de^	29.93 ± 0.40^ cd^	9.99 ± 0.26^ cd^	13 ± 0.25^b^
**T**_**5**_	44.7 ± 0.29^bcd^	318.91 ± 4.47^cde^	28.67 ± 0.57^bc^	18.36 ± 0.41^de^	17.37 ± 0.12^ cd^	31.82 ± 0.19^bc^	10.26 ± 0.38^ cd^	13.26 ± 0.08^b^
**T**_**6**_	44.63 ± 1.11^bcd^	322.8 ± 4.83^bcde^	29.18 ± 0.88^abc^	18.87 ± 0.86^cde^	17.88 ± 0.50^bcd^	32.09 ± 1.01^bc^	10.14 ± 0.30^ cd^	13.27 ± 0.39^b^
**T**_**7**_	46.04 ± 0.77^bc^	325.52 ± 8.38^bcde^	29.22 ± 0.50^abc^	19.9 ± 0.19^bcd^	18.41 ± 0.31^abc^	32.14 ± 0.55^bc^	10.91 ± 0.10^bc^	13.29 ± 0.13^b^
**T**_**8**_	46.61 ± 2.03^abc^	330.62 ± 6.99^abcde^	29.31 ± 1.23^abc^	19.97 ± 0.52^bcd^	18.94 ± 1.06^abc^	32.83 ± 1.35^b^	11.27 ± 0.45^ab^	13.67 ± 0.41^b^
**T**_**9**_	50.14 ± 0.41^a^	352.05 ± 4.65^a^	31.47 ± 0.47^a^	22.92 ± 0.18^a^	20.05 ± 0.16^a^	35.82 ± 0.37^a^	12.16 ± 0.10^a^	15.3 ± 0.12^a^
**T**_**10**_	47.88 ± 1.19^ab^	338.57 ± 2.81^abc^	30.12 ± 0.34^abc^	20.7 ± 0.71^b^	19.15 ± 0.24^ab^	33.96 ± 0.40^ab^	11.69 ± 0.17^ab^	13.82 ± 0.16^b^
**T**_**11**_	48.36 ± 0.25^ab^	341.97 ± 3.10^ab^	30.39 ± 0.26^ab^	20.91 ± 0.11^b^	19.34 ± 0.10^ab^	34.3 ± ^ab^0.17	11.8 ± 0.25^ab^	13.96 ± 0.07^b^
Mustard								
**T**_**1**_	20.68 ± 1.05^d^	239.3 ± 13.42^e^	31.01 ± ^c^1.04	9.76 ± 0.31^c^	41.25 ± 2.09^d^	30.33 ± 1.00^d^	9.8 ± 0.67^d^	11.2 ± 0.55^d^
**T**_**2**_	23.75 ± 0.49^abc^	270.11 ± 5.53^abcd^	35.61 ± 0.73^a^	11.21 ± 0.23^a^	47.37 ± 0.64^abc^	34.83 ± 0.71^ab^	11.25 ± 0.23^abc^	12.87 ± 0.26^ab^
**T**_**3**_	23.9 ± 0.76^ab^	271.73 ± 7.10^abc^	35.72 ± 0.84^a^	11.28 ± 0.23^a^	47.66 ± 1.56^ab^	35.04 ± 0.96^a^	11.32 ± 0.15^abc^	12.95 ± ^ab^0.20
**T**_**4**_	21.66 ± 0.54^ cd^	246.35 ± 6.10^de^	32.47 ± ^bc^0.85	10.23 ± 0.25^bc^	43.21 ± 1.07^ cd^	31.77 ± 0.79^ cd^	10.26 ± 0.25^ cd^	11.74 ± 0.29^ cd^
**T**_**5**_	21.71 ± 0.33^ cd^	246.83 ± 4.73^cde^	32.54 ± 0.67^bc^	10.25 ± 0.22^bc^	43.29 ± 0.47^ cd^	31.83 ± 0.61^ cd^	10.28 ± 0.34^ cd^	11.76 ± 0.08^ cd^
**T**_**6**_	21.92 ± 0.57^bcd^	249.28 ± 6.52^bcde^	32.86 ± 0.86^bc^	10.35 ± 0.27^bc^	43.72 ± ^bcd^1.14	32.15 ± 0.84^bcd^	10.39 ± 0.27^bcd^	11.88 ± 0.31^ cd^
**T**_**7**_	23.07 ± 0.21^abc^	262.83 ± 2.36^abcde^	34.96 ± 0.31^ab^	11.04 ± 0.10^ab^	46.16 ± 0.41^abc^	34.2 ± 0.30^abc^	10.83 ± 0.10^abcd^	12.53 ± 0.11^bc^
**T**_**8**_	23.13 ± 0.61^abc^	262.48 ± 6.91^abcde^	34.99 ± 0.91^ab^	10.95 ± 0.29^ab^	46.21 ± 1.21^abc^	34.18 ± 0.89^abc^	10.76 ± 0.29^abcd^	12.55 ± 0.33^bc^
**T**_**9**_	25.07 ± 0.20^a^	285.01 ± 2.29^a^	37.57 ± 0.30^a^	11.83 ± 0.10^a^	49.99 ± 0.40^a^	36.76 ± ^a^0.48	11.87 ± 0.10^a^	13.58 ± 0.11^a^
**T**_**10**_	24.09 ± 0.50^a^	273.88 ± 5.73^ab^	36.1 ± 0.76^a^	11.37 ± 0.24^a^	48.03 ± 1.01^a^	35.07 ± 0.74^a^	11.41 ± ^abc^0.24	13.05 ± 0.27^ab^
**T**_**11**_	24.32 ± 0.52^a^	276.52 ± 5.95^a^	36.45 ± 0.78^a^	11.48 ± 0.25^a^	48.5 ± 1.04^a^	35.66 ± 0.77^a^	11.52 ± ^ab^0.25	13.18 ± 0.14^ab^

Application of RDF + SNPs at the rate 3 mg S/kg soil (half at sowing and half as 1 MAS), resulted in a significantly higher seed and stover Fe content and uptake, with a value of 25.07, 285.01 ppm and 0.31 and 6.71 mg/pot, respectively, over remaining treatments barring RDF + ES at the rate 8 mg S/kg soil, RDF + ES at the rate 8 mg S/kg soil (half at sowing and half as 1 MAS), RDF + SNPs at the rate 4 mg S/kg soil and RDF + SNPs at the rate 4 mg S/kg soil (half at sowing and half as 1 MAS) for Fe content and uptake by seed and RDF + ES at the rate 8 mg S/kg soil, (half at sowing and half as 1 MAS), RDF + SNPs at the rate 4 mg S/kg soil and RDF + SNPs at the rate 4 mg S/kg soil (half at sowing and half as 1 MAS) for Fe content in stover and RDF + SNPs at the rate 4 mg S/kg soil and RDF + SNPs at the rate 4 mg S/kg soil (half at sowing and half as 1 MAS) in terms of Fe uptake by stover of mustard (Table 3). The control treatment, which consisted of pots treated solely with RDF, displayed a significantly lower seed and stover Fe content and uptake of 20.68, 239.30 ppm, 0.22 and 4.79 mg/pot, respectively. The Fe content and uptake by seed and stover increased 21.2, 19.1, 43.9 and 40.0% under RDF + SNPs at the rate 3 mg S/kg soil (half at sowing and half as 1 MAS) over control.

#### Zinc (Zn) content and uptake

**Fig. 3:**
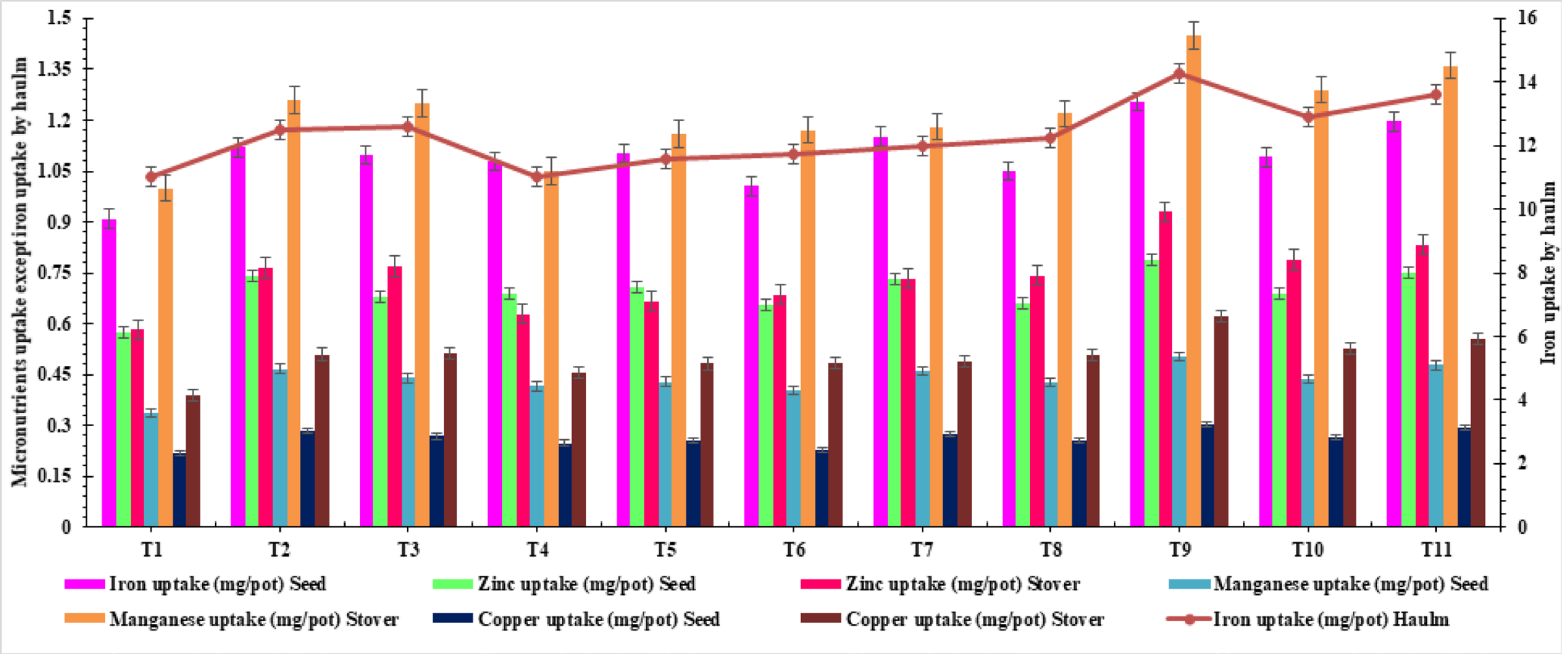
Effect of SNPs and ES on micronutrient uptake of groundnut.

**Fig. 4:**
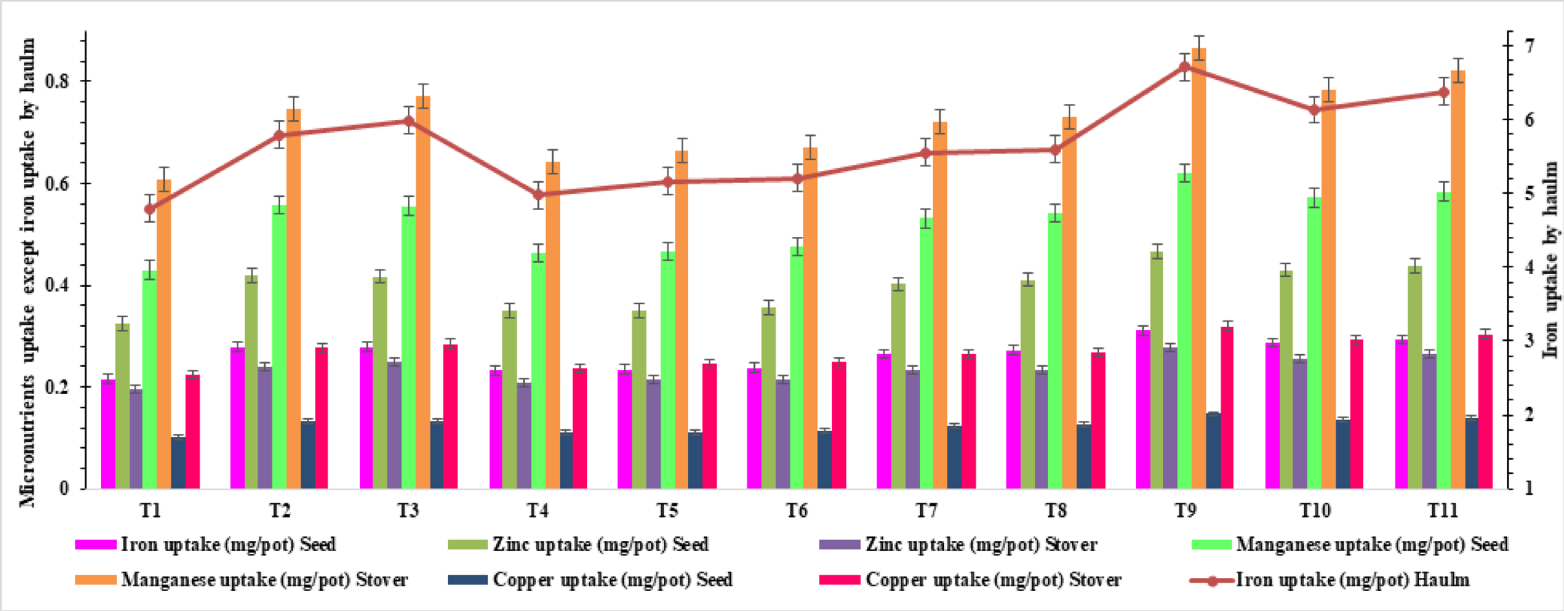
Effect of SNPs and ES on micronutrient uptake of mustard.

RDF with SNPs at the rate 3 mg S/kg soil (half at sowing and half as 1 MAS), resulted in significantly higher Zn content and uptake of seed and haulm, with a value of 31.4, 22.9 ppm and 0.79 and 0.93 mg/pot, respectively, than rest of the treatments except, RDF + ES at the rate 8 mg S/kg soil, RDF + SNPs at the rate 4 mg S/kg soil and RDF + SNPs at the rate 4 mg S/kg soil (half at sowing and half as 1 MAS) for Zn content in seed, while Zn content in haulm was significantly the higher under RDF + SNPs at the rate 3 mg S/kg soil (half at sowing and half as 1 MAS) and RDF + ES at the rate 8 mg S/kg soil, RDF + ES at the rate 8 mg S/kg soil (half at sowing and half as 1 MAS), RDF + SNPs at the rate 3 mg S/kg soil, RDF + SNPs at the rate 4 mg S/kg soil and RDF + SNPs at the rate 4 mg S/kg soil (half at sowing and half as 1 MAS) Zn uptake by seed and haulm. The Zn concentration and uptake by seed and haulm increased 20.7, 39.8, 36.2 and 60.34% higher under RDF + SNPs at the rate 3 mg S/kg soil (half at sowing and half as 1 MAS) over the control (Table 3, Figs. 3 and 4).

The application of RDF + SNPs at the rate 3 mg S/kg soil (half at sowing and half as 1 MAS), resulted in significantly higher seed and stover zinc content and uptake, with a value of 37.57, 11.83 ppm, 0.47 and 0.28 mg/pot respectively, over rest of the treatments except RDF + ES at the rate 8 mg S/kg soil, RDF + ES at the rate 8 mg S/kg soil (half at sowing and half as 1 MAS), RDF + SNPs at the rate 4 mg S/kg soil and RDF + SNPs at the rate 4 mg S/kg soil (half at sowing and half as 1 MAS) in terms of Zn content and RDF + SNPs at the rate 4 mg S/kg soil and RDF + SNPs at the rate 4 mg S/kg soil (half at sowing and half as 1 MAS) in terms of Zn uptake by seed and stover uptake significantly higher. The control treatment only RDF, displayed a significantly lower seed and stover zinc content and uptake of 31.01, 9.76 ppm, 0.32 and 0.20 mg/pot respectively (Table 3, Figs. 3 and 4). The Zn content and uptake by seed and stover under RDF + SNPs at the rate 3 mg S/kg soil (half at sowing and half as 1 MAS) was to the tune of 21.15, 21.21, 43.8 and 42.6% over control.

#### Manganese (Mn) content and uptake

RDF with SNPs at the rate 3 mg S/kg soil (half at sowing and half as 1 MAS) resulted in a significantly higher seed and haulm Mn content and uptake, with a value of 20.0, 35.8 ppm and 0.45 and 1.45 mg/pot, respectively (Table 3, Figs. 3 and 4) than other treatments barring RDF + ES at the rate 8 mg S/kg soil, RDF + ES at the rate 8 mg S/kg soil (half at sowing &half as 1 MAS), RDF + SNPs at the rate 3 mg S/kg soil, RDF + SNPs at the rate 4 mg S/kg soil for Mn content in seed and RDF + ES at the rate 8 mg S/kg soil, RDF + ES at the rate 8 mg S/kg soil (half at sowing and half as 1 MAS), RDF + SNPs at the rate 4 mg S/kg soil and RDF + SNPs at the rate 4 mg S/kg soil (half at sowing and half as 1 MAS) for Mn content in haulm and RDF + ES at the rate 8 mg S/kg soil, RDF + ES at the rate 8 mg S/kg soil (half at sowing and half as 1 MAS), RDF + SNPs at the rate 3 mg S/kg soil, RDF + SNPs at the rate 4 mg S/kg soil and RDF + SNPs at the rate 4 mg S/kg soil (half at sowing and half as 1 MAS) for Mn uptake by seed and RDF + SNPs at the rate 4 mg S/kg soil (half at sowing and half as 1 MAS) for Mn uptake by haulm of groundnut. Seed and haulm of groundnut contained 32.2, 26.8, 49.4 and 45.0% more Mn content and uptake respectively, under RDF + SNPs at the rate 3 mg S/kg soil (half at sowing and half as 1 MAS) than the control (Table 3, Figs. 3 and 4).

The fertilization of RDF + SNPs at the rate 3 mg S/kg soil (half at sowing and half as 1 MAS), resulted in a higher seed and stover Mn content and uptake, with values of 49.99, 36.76 ppm, 0.62 and 0.70 mg/pot, respectively, over rest of treatments except RDF + ES at the rate 8 mg S/kg soil, RDF + ES at the rate 8 mg S/kg soil (half at sowing and half as 1 MAS), RDF + SNPs at the rate 4 mg S/kg soil and RDF + SNPs at the rate 4 mg S/kg soil (half at sowing and half as 1 MAS) for Mn content in seed and stover and Mn uptake by seed while, RDF + SNPs at the rate 4 mg S/kg soil (half at sowing and half as 1 MAS) in terms of Mn uptake by stover of mustard. The control RDF showed a significantly lower seed and stover Mn content and uptake of 41.25, 30.33 ppm, 0.43 and 0.61 mg/pot respectively. The increase in Mn content and uptake by seed and stover of mustard was 21.2, 21.2, 44.2 and 42.5% higher with the application of RDF + SNPs at the rate 3 mg S/kg soil (half at sowing and half as 1 MAS) over control.

#### Copper (Cu) content and uptake

Application of RDF along with SNPs at the rate 3 mg S/kg soil (half at sowing and half as 1 MAS) resulted in higher Cu content and uptake in the seed and haulm, with values of 12.1, 15.3 ppm, 0.30 and 0.62 mg/pot respectively, than other treatments, while it was on par with RDF + ES at the rate 8 mg S/kg soil, RDF + ES at the rate 8 mg S/kg soil (half at sowing and half as 1 MAS), RDF + SNPs at the rate 4 mg S/kg soil and RDF + SNPs at the rate 4 mg S/kg soil (half at sowing and half as 1 MAS) for Cu content in seed, for haulm it was significantly superior. The control RDF showed the lowest Cu content and uptake of Cu in seed and haulm of groundnut, with a value of 9.79, 10.9 ppm and 0.22 and 0.39 mg/pot, respectively. The percentage increase of Cu content and uptake by seed and haulm of groundnut was 24.2, 39.8, 36.3 and 58.9% respectively, with RDF + SNPs at the rate 3 mg S/kg soil (half at sowing and half as 1 MAS) over control (Table 3).

At RDF + SNPs at the rate 3 mg S/kg soil (half at sowing and half as 1 MAS), resulted in the higher Cu content and uptake in the seed and stover of mustard, with values of 11.87, 13.58 ppm, 0.15 and 0.32 mg/pot, respectively over rest of the treatments except RDF + ES at the rate 8 mg S/kg soil, RDF + ES at the rate 8 mg S/kg soil (half at sowing and half as 1 MAS), RDF + SNPs at the rate 4 mg S/kg soil and RDF + SNPs at the rate 4 mg S/kg soil (half at sowing and half as 1 MAS) in terms of Cu concentration in seed and stover while Cu uptake being at par with RDF + ES at the rate 8 mg S/kg soil, RDF + ES at the rate 8 mg S/kg soil (half at sowing and half as 1 MAS), RDF + SNPs at the rate 4 mg S/kg soil and RDF + SNPs at the rate 4 mg S/kg soil (half at sowing and half as 1 MAS) in case of seed while in case of stover RDF + SNPs at the rate 4 mg S/kg soil (half at sowing and half as 1 MAS) found on par. The control only RDF showed a significantly lower Cu content in seed and stover, with a value of 9.80, 11.20 ppm, 0.10 and 0.22 mg/pot respectively. The increase in Cu content and uptake in seed and stover with application of RDF + SNPs at the rate 3 mg S/kg soil (half at sowing and half as 1 MAS) was to the tune of 21.1, 21.2, 44.1 and 42.8% higher over control (Table 3, Figs. 3 and 4).

### Sulphur dynamics

Significantly higher water soluble, heat soluble, organic, adsorbed sulphate sulphur with the value of 3.17, 5.48, 38.14 and 5.42 mg/kg, respectively (Table 4) was obtained under application of RDF + sulphur nanoparticles at the rate 4.0 mg S/kg soil, (half at sowing and half as 1 MAS) than the rest of the treatments barring RDF + elemental S at the rate 8.0 mg S/kg soil, RDF + elemental S at the rate 8.0 mg S/kg soil, (half at sowing and half as 1 MAS), RDF + sulphur nanoparticles at the rate 3.0 mg S/kg soil, (half at sowing and half as 1 MAS) and RDF + sulphur nanoparticles at the rate 4.0 mg S/kg soil for heat soluble, adsorbed sulphate and water soluble sulphur and RDF + elemental S at the rate 8.0 mg S/kg soil, RDF + sulphur nanoparticles at the rate 3.0 mg S/kg soil, (half at sowing and half as 1 MAS) and RDF + sulphur nanoparticles at the rate 4.0 mg S/kg soil for organic sulphur. The numerically lowest water, heat soluble, organic and adsorbed sulphate sulphur with the value of 1.97, 4.25, 35.5, and 3.44 mg/kg, respectively were obtained in the control treatment which didn’t receive sulphur throughout the cropping.

**Table 4:** Effect of SNPs and elemental sulphur on fractionation in soil after harvest of groundnut-mustard sequence (After harvest of Mustard).

Treatments	Sulphur fractions (mg /kg)					
	Heat soluble	Total sulphur	Organic sulphur	Adsorbed sulphate sulphur	Water soluble sulphur	Non-sulphate sulphur
**T**_**1**_	4.25 ± 0.06^d^	56.65 ± 0.97^b^	35.5 ± 0.25^abc^	3.44 ± 0.05^e^	1.97 ± 0.037f.	17.7 ± 0.71^a^
**T**_**2**_	5.05 ± 0.22^abc^	61.18 ± 1.33^ab^	37.63 ± 1.27^ab^	5.03 ± 0.15^a^	3.02 ± 0.024^ab^	18.53 ± 1.11^a^
**T**_**3**_	5.1 ± 0.07^ab^	60.85 ± 0.50^ab^	36.98 ± 0.29^abc^	4.97 ± 0.04^ab^	3.00 ± 0.184^ab^	18.89 ± 0.62^a^
**T**_**4**_	4.29 ± 0.25^d^	57.49 ± 0.38^ab^	34.31 ± 0.60^c^	3.92 ± 0.20^de^	2.36 ± 0.034^e^	19.26 ± 0.49^a^
**T**_**5**_	4.46 ± 0.07^d^	58.54 ± 2.05^ab^	35.28 ± 0.37^bc^	3.95 ± 0.24^de^	2.54 ± 0.029^de^	19.31 ± 2.35^a^
**T**_**6**_	4.53 ± 0.06^d^	58.59 ± 0.50^ab^	35.5 ± 0.53^abc^	4.24 ± 0.14^ cd^	2.58 ± 0.017^de^	18.86 ± 0.36^a^
**T**_**7**_	4.62 ± 0.08^ cd^	60.39 ± 0.54^ab^	35.81 ± 1.09^abc^	4.4 ± 0.15^ cd^	2.63 ± 0.044^ cd^	20.18 ± 1.39^a^
**T**_**8**_	4.72 ± 0.07^bcd^	60.71 ± 0.22^ab^	35.81 ± 0.59^abc^	4.5 ± 0.03^bc^	2.83 ± 0.031^bc^	20.4 ± 0.70^a^
**T**_**9**_	5.4 ± 0.21^a^	61.51 ± 1.20^ab^	37.64 ± 0.43^ab^	5.2 ± 0.17^a^	3.14 ± 0.017^a^	18.67 ± 1.40^a^
**T**_**10**_	5.18 ± 0.07^ab^	61.06 ± 0.27^ab^	37.25 ± 0.25^ab^	5.08 ± 0.20^a^	3.01 ± 0.108^ab^	18.73 ± 0.20^a^
**T**_**11**_	5.48 ± 0.08^a^	61.89 ± 3.12^a^	38.14 ± 1.02^a^	5.42 ± 0.11^a^	3.17 ± 0.046^a^	18.33 ± 3.16^a^

Highest non-sulphate sulphur was found with the value of 20.4 mg/kg with application of RDF + sulphur nanoparticles at the rate 3.0 mg S/kg soil. Moreover, the highest total sulphur of 61.89 mg/kg was registered with the application of RDF + sulphur nanoparticles at the rate 4.0 mg S/kg soil, (half at sowing and half as 1 MAS) followed by 61.51 mg/kg was obtained with RDF + sulphur nanoparticles at the rate 3.0 mg S/kg soil, (half at sowing and half as 1 MAS). Wherever, the lowest non-sulphate and total sulphur fraction was measured under control treatment with the value of 17.7 and 56.65 mg/kg.

### DTPA extractable micronutrients in soil after harvest of groundnut and mustard

Available zinc content in soil at harvest of mustard varies from 0.60 to 0.67 ppm, with the highest value 0.667 mg/kg was obtained under RDF + SNPs at the rate 4 mg S/kg soil (half at sowing and half as 1 MAS) (Table 5). The available iron, manganese and copper in the soil at harvest vary from 4.19 to 4.63 ppm, 5.81 to 6.43 ppm and 1.03 to 1.14 ppm, respectively. Higher micronutrients 4.63, 6.43 and 1.14 mg/kg (Fe, Mn and Cu) respectively, were found under RDF + SNPs at the rate 4 mg S/kg soil (half at sowing and half as 1 MAS).

**Table 5:** Effect of SNPs and ES on DTPA-extractable micronutrients in soil after harvest of groundnut-mustard.

Treatments	DTPA-extractable micronutrients (ppm)			
	Iron	Zinc	Manganese	Copper
**T**_**1**_	4.19 ± 0.13^b^	0.6 ± 0.054^b^	5.81 ± 0.16^b^	1.03 ± 0.020^b^
**T**_**2**_	4.4 ± 0.24^ab^	0.634 ± 0.051^ab^	6.11 ± 0.27^ab^	1.08 ± 0.045^ab^
**T**_**3**_	4.43 ± 0.08^ab^	0.638 ± 0.049^ab^	6.15 ± 0.11^ab^	1.09 ± 0.019^ab^
**T**_**4**_	4.2 ± 0.12^b^	0.608 ± 0.053^b^	5.83 ± 0.17^b^	1.03 ± 0.031^b^
**T**_**5**_	4.19 ± 0.06^b^	0.608 ± 0.053^b^	5.82 ± 0.08^b^	1.03 ± 0.027^b^
**T**_**6**_	4.21 ± 0.03^ab^	0.61 ± 0.053^b^	5.85 ± 0.05^b^	1.04 ± 0.009^b^
**T**_**7**_	4.35 ± 0.01^ab^	0.629 ± 0.052^ab^	6.04 ± 0.01^ab^	1.07 ± 0.002^ab^
**T**_**8**_	4.35 ± 0.10^ab^	0.63 ± 0.052^ab^	6.04 ± 0.14^ab^	1.07 ± 0.024^ab^
**T**_**9**_	4.47 ± 0.11^ab^	0.646 ± 0.048^ab^	6.21 ± 0.15^ab^	1.1 ± 0.027^ab^
**T**_**10**_	4.5 ± 0.05^ab^	0.649 ± 0.046^ab^	6.25 ± 0.07^ab^	1.11 ± 0.012^ab^
**T**_**11**_	4.63 ± 0.10^a^	0.667 ± 0.046^a^	6.43 ± 0.13^a^	1.14 ± 0.023^a^

### Sulphur use efficiency

Significantly higher sulphur utilization efficacy 121.37 and 91.30%, was registered with the application of RDF + sulphur nanoparticles at the rate 1.0 mg S/kg soil, (half at sowing and half as 1 MAS) and RDF + sulphur nanoparticles at the rate 3.0 mg S/kg soil, (half at sowing and half as 1 MAS) over the rest of the treatments. Moreover, the lowest sulphur utilization efficacy was measured under control with the values 19.28 and 18.51% under RDF + elemental sulphur 8 mg/kg soil. It was also observed that the sulphur nano particles have more utilization efficacy than the elemental sulphur. The sulphur content and uptake were significantly higher so, the sulphur use efficiency was much higher in RDF + sulphur nanoparticles at the rate 3.0 mg S/kg soil, (half at sowing and half as 1 MAS).

### Pearson’s correlation

Among various sulphur fractions (heat soluble sulphur, total sulphur, organic sulphur, adsorbed sulphate, water soluble sulphur, and non-sulphate) with crop yields (specifically groundnut pod yield, groundnut haulm yield, mustard grain yield, and mustard straw yield) (Table 6). Positive correlation values, having R^2^ = 0.91 to R^2^ = 0.97, revealed robust positive associations, implying that there is a tendency for crop yields to increase as sulphur percentages increase. It is worth mentioning that there is a notable positive association between groundnut pod yield (GPY) and groundnut haulm yield (GHY) with various sulphur fractions, suggesting a major favourable impact. In contrast, it can be shown that non-sulphate sulphur (NS-S) exhibits a negative correlation with both total sulphur and organic sulphur, indicating an inverse association between these variables. This information facilitates comprehension of the influence of various sulphur fractions on agricultural productivity within the framework of the provided dataset.

**Table 6:** Relationship between sulphur fractions and yield using Pearson’s correlation.

Parameters	*HS-S*	*TS*	*OS*	*Ads S*	*WS*	*NS-S*	GPY	GHY	MGY
TS	0.91**	1.00**							
OS	0.95**	0.85**	1.00**						
Ads S	0.97**	0.95**	0.90**	1.00**					
WS	0.94**	0.96**	0.84**	0.98**	1.00**				
NS-S	− 0.21	0.16**	− 0.37**	− 0.07	0.07**	1.00**			
GPY	0.26**	0.30**	0.18**	0.30**	0.32**	0.16**	1.00**		
GHY	0.93**	0.83**	0.86**	0.87**	0.84**	− 0.16	0.32**	1.00**	
MGY	0.93**	0.96**	0.85**	0.94**	0.93**	0.08**	0.28**	0.90**	1.00**
MSY	0.96**	0.86**	0.87**	0.91**	0.89**	− 0.14	0.28**	0.98**	0.91**

(HS: Heat soluble, TS: Total sulphur, OS: organic sulphur, Ads. S: Adsorbed sulphate sulphur, WS: water soluble sulphur, NS-S: non-sulphate sulphur, GPY: Groundnut pod yield, GHY: Groundnut haulm yield, MGY: Mustard grain yield, MSY: Mustard straw yield).

### Discussion

Sulphur is a crucial element for the growth and development of plants, as it is required for the synthesis of proteins, activation of enzymes, and the production of chlorophyll and it play a vital role in many physiological and biochemical processes within plants^53–56^. The sulphur plays an important role in the formation of chlorophyll that directly involves in photosynthesis. Chlorophyll plays a vital role in plants, serving as the pigment responsible for their green colour. Its importance lies in the process of photosynthesis, where plants convert sunlight into biochemical energy. Similar positive effect of sulphur nano particles was reported^57,58^, who observed that application of sulphur nanoparticles increased chlorophyll content and photosynthetic rate in mustard plants.

Significantly higher pods and siliqua/plant were found due to the application of sulphur. The application of RDF + sulphur nanoparticles at 3.0 mg S/kg soil, (half at sowing and half as 1 MAS) can be an optimum dose of sulphur through sulphur nano particles. Due to the optimum dose of sulphur as well as a split application, it was being available throughout the entire crop growth period that improves vegetative growth and development of both crops^59^. Findings found that the application of SNPs showed a significant increase in branches, pods per plant and yield of groundnut over control^60–62^.

**Fig. 5:**
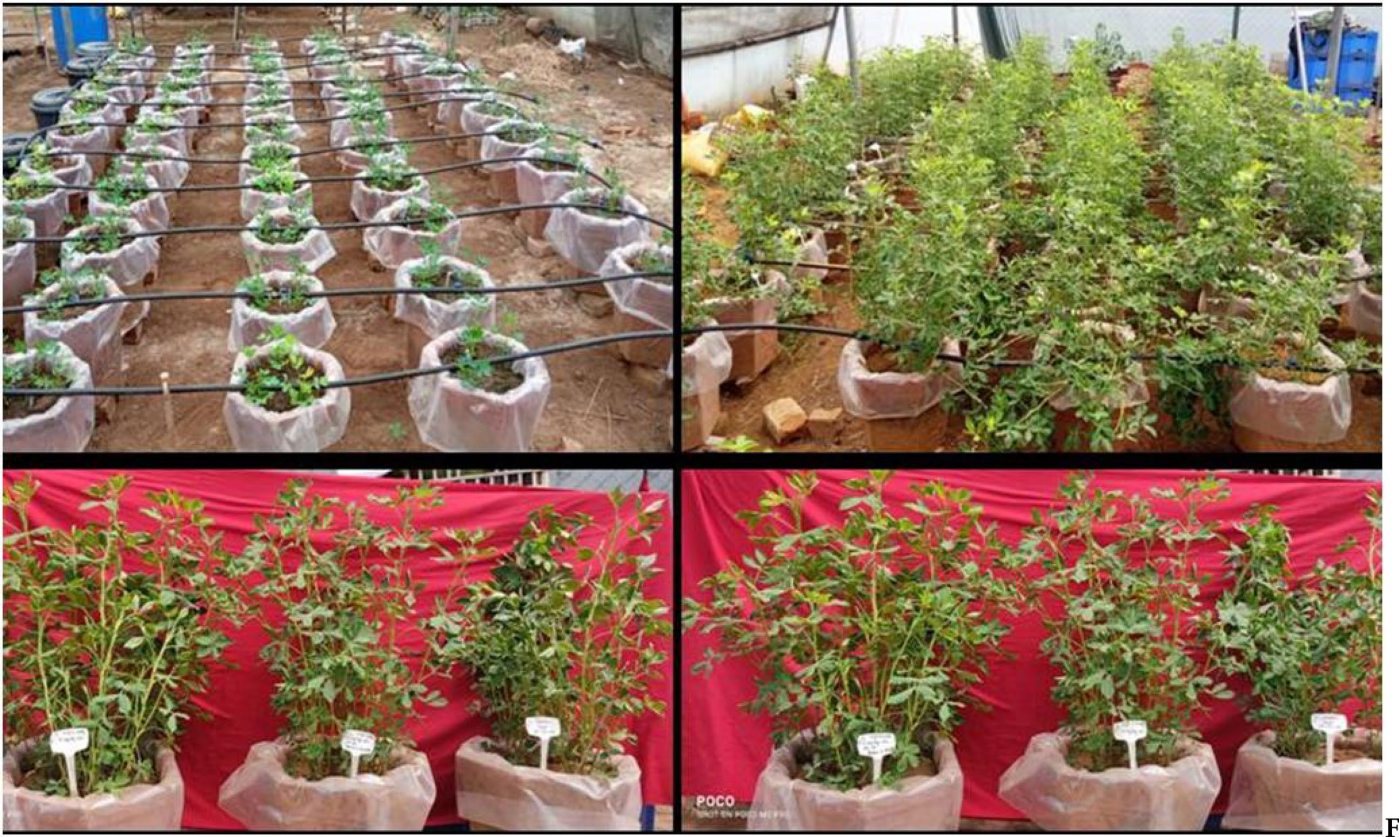
Overview and different growth stages of groundnut.

**Fig. 6:**
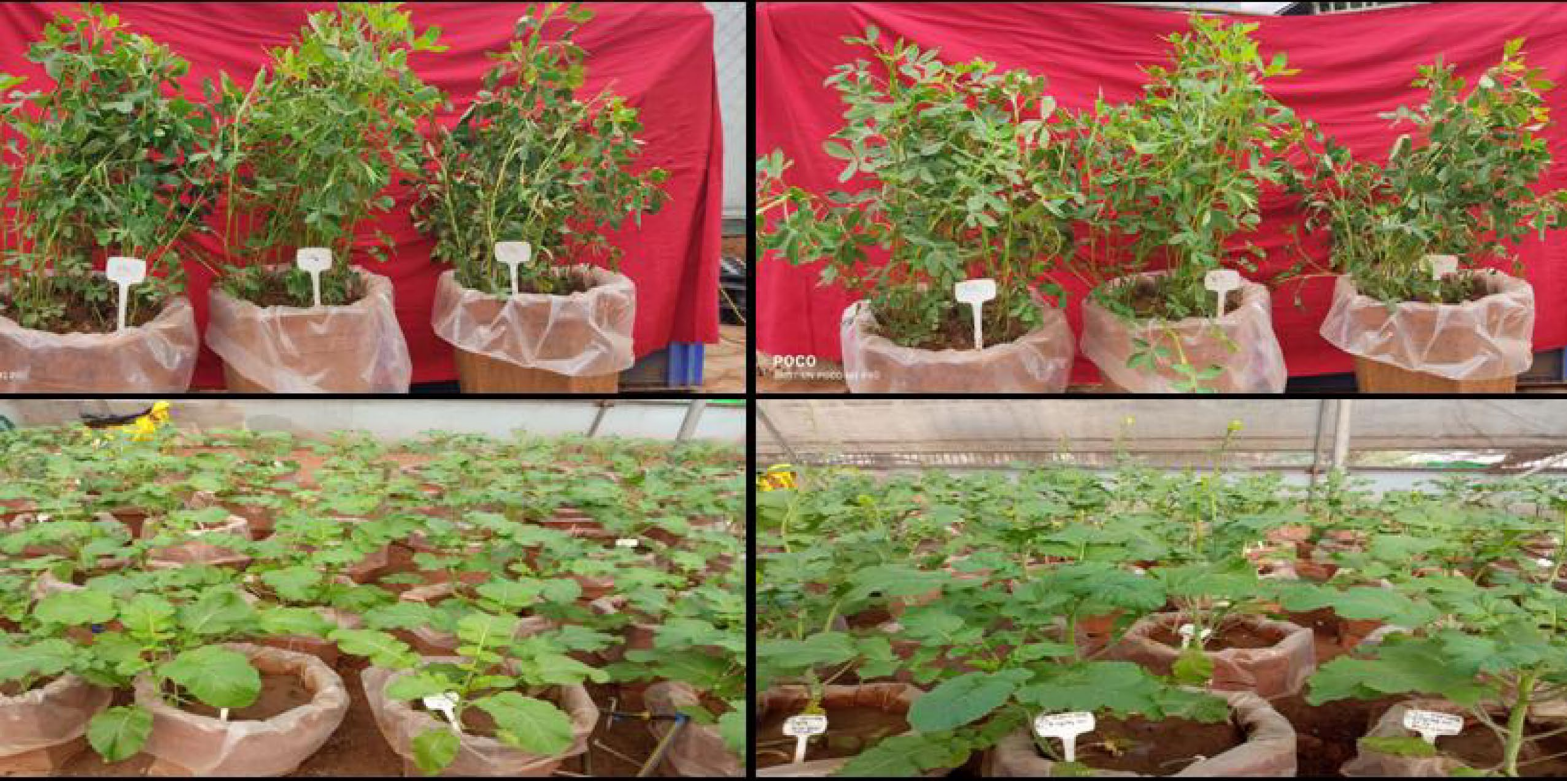
Growth stages of groundnut and overview of mustard crop.

**Fig. 7:**
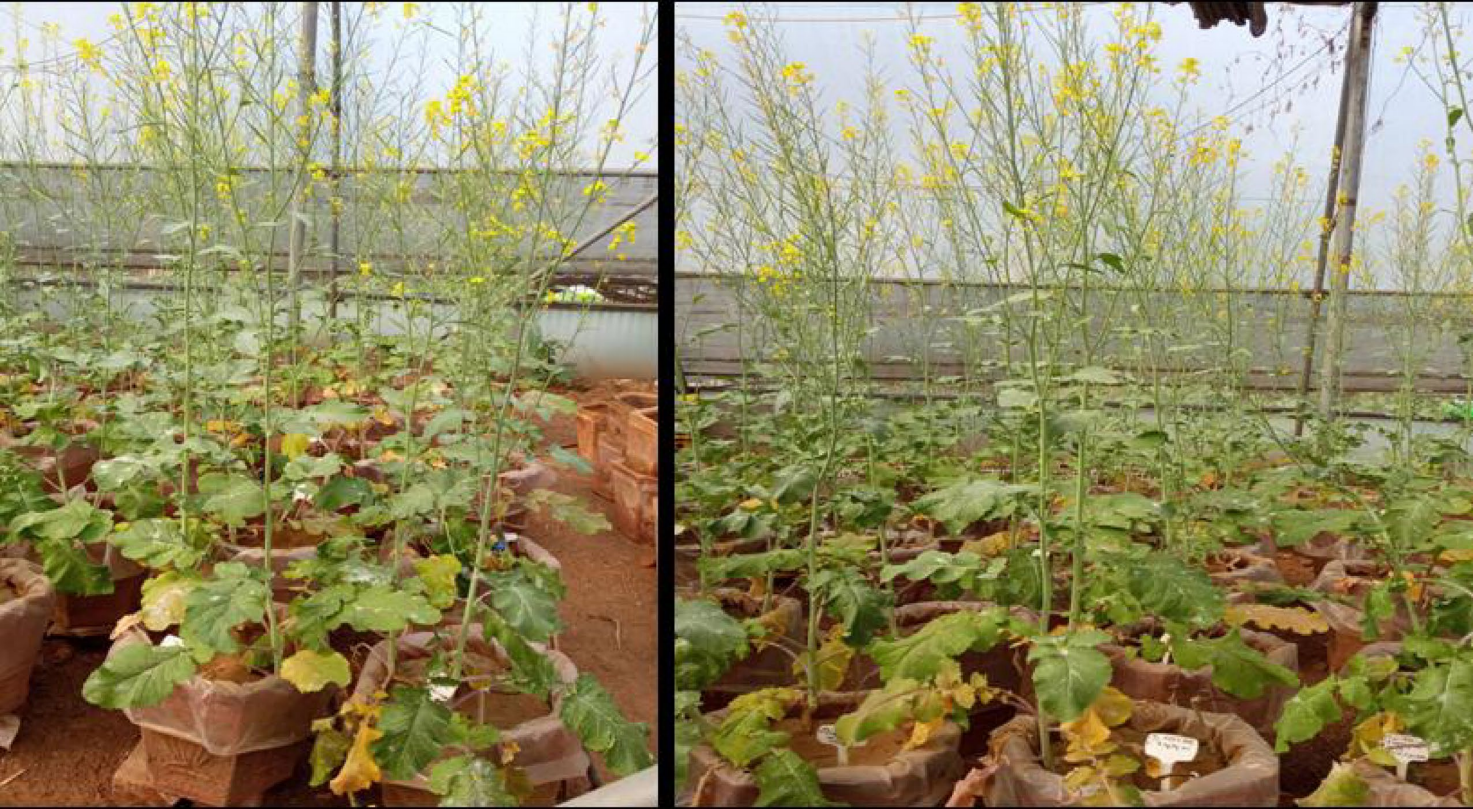
Pod formation stage of mustard.

These sulphur sources help to fulfil the sulphur requirements of the plants, leading to increase sulphur content in different plant parts as well as increase transformation and metabolism of sulphur in different plant parts (Fig. 5). Also, sulphur application improves the activity of sulphur related enzymes and transporters involved in sulphur uptake, assimilation and redistribution, thereby increasing the sulphur content in different plant parts (Fig. 6). Similarly, reported that the application of SNPs significantly increased the sulphur content of maize kernels and leaves by 20.6 and 36.8%, respectively^63^, compared to control (Fig. 7). These findings align with recent studies emphasizing the importance of integrating nutrient dynamics^64^, biochar amendments, and rhizosphere interactions to enhance nutrient use efficiency and stress resilience in cropping systems^65,66^.

The application of SNPs and elemental sulphur as fertilizers can increase the sulphur uptake in both seed and haulm of groundnut and mustard plants. This is primarily due to enhanced sulphur availability, improved nutrient uptake, efficiency, and the facilitation of sulphur assimilation and transport within the plant (Figs. 5, 6 and 7). Thereby, plants assimilate more of the sulphur in different plant parts that’s why increased the plant uptake of sulphur. Rajput et al.^67^ and Karimi et al.^68^, documented analogous outcome, observing that the utilization of sulphur led to enhanced uptake of sulphur across various plant parts. RDF + sulphur nanoparticles at the rate 3.0 mg S/kg soil, (half at sowing and half as 1 MAS) have balanced nutrition of other essential nutrients for the groundnut and mustard, so the growth and yield parameters were more. So, obtained the highest sulphur uptake and use efficiency.

The content and uptake of micronutrients (Zn, Mn, Fe, and Cu) were considerably influenced by the application of sulphur. With the application of sulphur to soil, it may change the pH, solubility, microbial activity, and encourage root development which may affect the availability and uptake of micronutrients. Thereby, the availability of cationic micronutrients is affected by pH change. Higher micronutrient concentrations in plant tissues may come from better micronutrient uptake as a result of this^69,70^. As well as the experimental soil has neutral reaction that enhances the nutrients absorption. Sulphur can combine with specific micronutrients to generate complexes that increase their availability and stop them from precipitating in the soil. This makes it easier for plant roots to absorb them.

Numerous controllable and uncontrollable factors affect the growth and development of the plant, and one of the most important of these is well-balanced nutrition. Ferredoxin and acetyl-CoA have sulphur and are essential for the reduction of CO_2_ and the synthesis of organic molecules, the availability of sulphur may have an impact on the rate of photosynthetic^71^. The beneficial effects of RDF and S application on root growth and morphology have been demonstrated in several studies^72–75^.

Sulphur, for instance, has an impact on the production of certain molecules involved in the transport and absorption of nutrients^76^. The application of sulphur can modify the microbial populations in soil, which in turn can have an impact on nutrient availability and cycling^77^. Some soil bacteria help to solubilize and mobilise micronutrients^78–80^, which has an impact on how well plants absorb those minerals^81,82^.

The numerically lowest water, heat soluble, organic and adsorbed sulphate sulphur were obtained in the control treatment which didn’t receive sulphur throughout the cropping. The organic sulphur fraction was the dominant form of sulphur in soil. The variation in the organic sulphur is mainly due to mineralization and oxidation of sulphur and is also based on organic carbon content and clay fraction of soil. Adsorbed sulphur was extracted from the exchange complex of the soil by the extractant. The water soluble and adsorbed sulphur fractions were increased in the soil and it is mainly due to slow and steady releasing behaviour of the nano based fertilizer. Occluded sulphur or non-sulphate sulphur is mostly made up of sulphate occluded in and adsorbed on carbonates or insoluble sulphur compounds of iron and aluminium in soil which remains unextractable after removal of organic carbon and sulphate sulphur. The total sulphur content of the soil was increased with an increase in organic carbon content and finer fraction of the soil, while less affected by lower doses of sulphur application^83–87^. The findings unequivocally demonstrate that, while having no discernible impact, the smallest particle size of S increased the amount of DTPA extractable micronutrients in the soil relative to the baseline measurements^88–94^.

### Conclusion

**Fig. 8:**
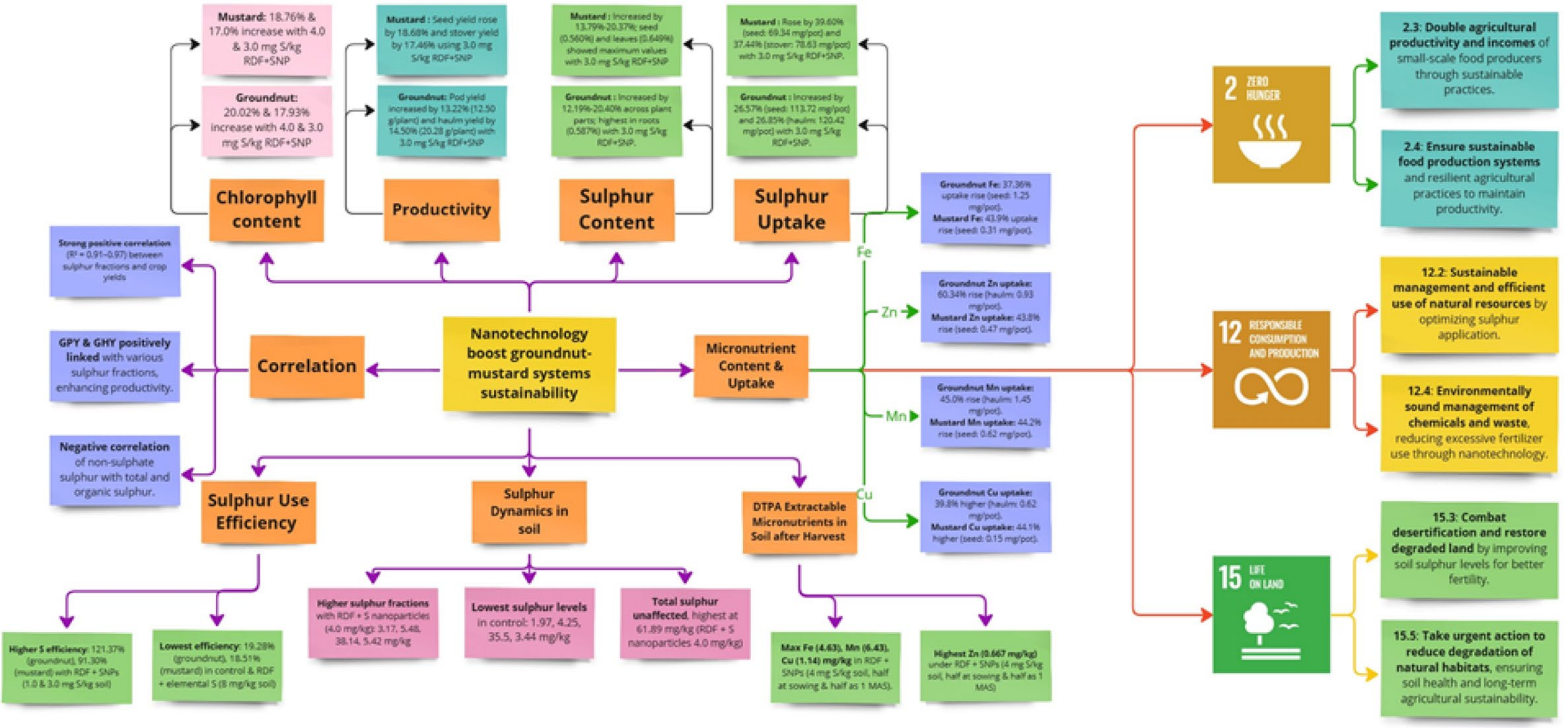
Nanotechnology boost groundnut-mustard systems sustainability and relevant SGDs.

This study highlights the transformative potential of sulphur nanoparticles in enhancing crop productivity and soil nutrient dynamics (Fig. 8). The optimized application of SNPs significantly improved yield, nutrient uptake, and sulphur use efficiency in the groundnut-mustard system, offering a sustainable alternative to conventional sulphur fertilizers. Results suggest that application of RDF + sulphur nanoparticles at 3.0 mg S/kg soil, (half at sowing and half at 1 MAS) can be an optimum dose of sulphur for groundnut and mustard crops under controlled environment of pot experiment. The higher chlorophyll content, yield, nutrients content and uptake were obtained with the application of RDF + sulphur nanoparticles at the rate 3.0 mg S/kg soil, (half at sowing and half as 1 MAS), while the higher sulphur fractionation and DTPA extractable micronutrients in soil were obtained under RDF + sulphur nanoparticles at the rate 4 mg S/kg soil, (half at sowing and half as 1 MAS). Based on the findings, the recommended strategy for optimal sulphur management in sulphur-deficient soils is the application of RDF + sulphur nanoparticles (SNPs) at 3.0 mg S/kg soil, applied in a split dose (half at sowing and half at 1 MAS). This approach significantly enhances yield, nutrient uptake, and sulphur use efficiency in the groundnut-mustard cropping system. Additionally, higher sulphur fractions and DTPA extractable micronutrients were observed at 4.0 mg S/kg SNPs, indicating its role in improving soil fertility. The use of SNPs reduces reliance on conventional fertilizers, promoting sustainable and efficient nutrient management in modern agriculture.

## Supplementary Information

Below is the link to the electronic supplementary material.

Supplementary Material 1

## Data Availability

Data will be available upon reasonable request from the corresponding author as well as the first author.

## References

[1] Gupta, G. et al. Microbes-mediated integrated nutrient management for improved rhizo-modulation, pigeonpea productivity, and soil bio-fertility in a semi-arid agro-ecology. *Front. Microbio.* **13**, 924407. 10.3389/fmicb.2022.924407 (2022). 10.3389/fmicb.2022.924407 PMC9520524 36187978

[2] Kumar, L. M. & Indira, M. Trends in fertilizer consumption and foodgrain production in India: A co-integration analysis#. *SDMIMD J. Manag.* **8**, 45–50. 10.18311/sdmimd/2017/18025 (2017).

[3] Gupta, G. et al. Assessment of bio-inoculants-mediated nutrient management in terms of productivity, profitability and nutrient harvest index of pigeon pea–wheat cropping system in India. *J. Plant Nutr.* **43**, 2911–2928 (2020).

[4] Rashmi, I. et al. Gypsum-an inexpensive, effective sulphur source with multitude impact on oilseed production and soil quality-A review. *Agric. Rev.* **39**, 218–225. 10.18805/ag.R-1792 (2018).

[5] Joshi, N., Gothalwal, R., Singh, M. & Dave, K. Novel sulphur-oxidizing bacteria consummate sulphur deficiency in oil seed crop. *Arch. Microbio.* **203**, 1–6. 10.1007/s00203-020-02009-4 (2021). 10.1007/s00203-020-02009-4 32757115

[6] Zenda, T., Liu, S., Dong, A. & Duan, H. Revisiting sulphur—The once neglected nutrient: It’s roles in plant growth, metabolism, stress tolerance and crop production. *Agriculture* **11**, 626. 10.3390/agriculture11070626 (2021).

[7] Solaimalai, A., Jayakumar, M., Baskar, K. & Senthilkumar, M. Sulphur fertilization in groundnut crop in India: A review. *Ind. Soc. Oilseeds Res.* **37**, 1–10 (2020).

[8] Ihsan MZ, Daur I, Alghabari F, Alzamanan S, Rizwan S, Ahmad M, Shafqat W (2019) Heat stress and plant development: role of sulphur metabolites and management strategies. Acta AgriculturaeScandinavica, Section B—Soil and Plant Science 69: 332–342.10.1080/09064710.2019.1569715.

[9] Lajoie-O’Malley, A., Bronson, K., Van der Burg, S. & Klerkx, L. The future (s) of digital agriculture and sustainable food systems: An analysis of high-level policy documents. *Ecosystem Ser.* **45**, 101183. 10.1016/j.ecoser.2020.101183 (2020).

[10] Struik, P. C. & Kuyper, T. W. Sustainable intensification in agriculture: the richer shade of green A review. *Agron. Sustain. Dev.* **37**, 1–15. 10.1007/s13593-017-0445-7 (2017).

[11] Saritha, G. N. G., Anju, T. & Kumar, A. Nanotechnology-Big impact: How nanotechnology is changing the future of agriculture?. *J. Agri. Food Res.* **10**, 1–19. 10.1016/j.jafr.2022.100457 (2022).

[12] Sharma, B., Tiwari, S., Kumawat, K. C. & Cardinale, M. Nano-biofertilizers as bio-emerging strategies for sustainable agriculture development: Potentiality and their limitations. *Sci. Total Environ.* **860**, 160476. 10.1016/j.scitotenv.2022.160476 (2023). 36436627 10.1016/j.scitotenv.2022.160476

[13] Ghormade, V., Deshpande, M. V. & Paknikar, K. M. Perspectives for nano-biotechnology enabled protection and nutrition of plants. *Biotech. Adva* **29**, 792–803. 10.1016/j.biotechadv.2011.06.007 (2011). 10.1016/j.biotechadv.2011.06.007 21729746

[14] Manjunatha, S. B., Biradar, D. P. & Aladakatti, Y. R. Nanotechnology and its applications in agriculture: A review. *J. Farm. Sci.* **29**, 1–13 (2016).

[15] Dimkpa, C. O. & Bindraban, P. S. Nanofertilizers: New products for the industry. *J. Agril. Food Chem.* **66**, 6462–6473. 10.1021/acs.jafc.7b02150 (2017). 10.1021/acs.jafc.7b02150 28535672

[16] Kanjana D (2017) Advancement of Nanotechnology Applications on Plant Nutrients Management and Soil Improvement. In: Prasad, R., Kumar, V., Kumar, M. (eds) Nanotechnology. Springer, Singapore. 10.1007/978-981-10-4678-0_12.

[17] Subramanian KS, Thirunavukkarasu M (2017) Nano-fertilizers and Nutrient Transformations in Soil. In: Ghorbanpour, M., Manika, K., Varma, A. (eds) Nanoscience and Plant–Soil Systems. Soil Biology, 305–319. Springer, Cham. 10.1007/978-3-319-46835-8_11.

[18] Vejan, P., Khadiran, T., Abdullah, R. & Ahmad, N. Controlled release fertilizer: A review on developments, applications and potential in agriculture. *J. Contrel.* **339**, 321–334. 10.1016/j.jconrel.2021.10.003 (2021). 10.1016/j.jconrel.2021.10.003 34626724

[19] Qureshi, A., Singh, D. K. & Dwivedi, S. Nano-fertilizers: A novel way for enhancing nutrient use efficiency and crop productivity. *Int. J. Curr. Microbio. App. Sci.* **7**, 3325–3335 (2018).

[20] Yadav, A., Yadav, K. & Abd-Elsalam, K. A. Nanofertilizers: Types, delivery and advantages in agricultural sustainability. *Agrochem.* **2**, 296–336. 10.3390/agrochemicals2020019 (2023).

[21] Mukhopadhyay, S. S. Nanotechnology in agriculture: Prospects and constraints. *Nanotechn. Sci. Appl.* **7**, 63–71. 10.2147/NSA.S39409 (2014). 10.2147/NSA.S39409 PMC4130717 25187699

[22] Seleiman MF, Hafez EM (2021) Optimizing inputs management for sustainable agricultural development. Mitigating environmental stresses for agricultural sustainability in Egypt, 487–507.10.1007/978-3-030-64323-2_18.

[23] Duhan, J. S. et al. Nanotechnology: The new perspective in precision agriculture. *Biotech. Rep.* **15**, 11–23. 10.1016/j.btre.2017.03.002 (2017). 10.1016/j.btre.2017.03.002 PMC5454086 28603692

[24] Iqbal, M. A. Nano-fertilizers for sustainable crop production under changing climate: A global perspective. *SustainCropProd* **8**, 1–13 (2019).

[25] Mali, S. C., Raj, S. & Trivedi, R. Nanotechnology a novel approach to enhance crop productivity. *Biochem. Biophys. Rep.* **24**, 100821. 10.1016/j.bbrep.2020.100821 (2020). 33015378 10.1016/j.bbrep.2020.100821 PMC7522746

[26] Smartt J (Ed.) (2012) The groundnut crop: A scientific basis for improvement. Springer Science and Business Media.

[27] Stalker HT, Tallury SP, Seijo GR, Leal-Bertioli SC (2016) Biology, speciation, and utilization of peanut species. In Peanuts (pp. 27–66). AOCS Press.

[28] Anonymous (2023) The Solvent Extractors Association of India. Ag Statistics Web. https://www.indiastat.com/table/foreign-trade/commodity-wise-export-oilmeals-india-1997-1998-202/101396#. Accessed 26 June 2023.

[29] Rai PK, Yadav P, Kumar A, Sharma A, Kumar V, Rai P (2022) Brassica juncea: A crop for food and health. In The Brassica juncea Genome (pp. 1–13). Cham: Springer International Publishing.10.1007/978-3-030-91507-0_1.

[30] Nair KP, Nair KP (2021) Mustard. Minor Spices and Condiments: Global Economic Potential, 89–97.10.1007/978-3-030-82246-0_10.

[31] Francisco, M. et al. Nutritional and phytochemical value of Brassica crops from the agri-food perspective. *Ann. App. Bio.* **170**, 273–285. 10.1111/aab.12318 (2017).

[32] Sharma, R. K., Cox, M. S., Oglesby, C. & Dhillon, J. S. Revisiting the role of sulfur in crop production: A narrative review. *J. Agric. Food Res.* **15**, 101013. 10.1016/j.jafr.2024.101013 (2024).

[33] Kalyani, R., Nagavani, A. V. & Mobeena, S. Effect of varieties and graded levels of sulphur on yield, quality and economics of summer sesame. *Agric. Sci. Digest* **45**, 257. 10.18805/ag.D-5597 (2025).

[34] Dhumgond, P., Laxmanarayanan, M., Jahir Basha, C. R. & Prakash, N. B. Effect of different rate and time of application of slag-based gypsum on nutrient use efficiency, quality and yield of groundnut. *Legume Res.* **48**, 975. 10.18805/LR-4981 (2025).

[35] Wahane, M. R., Salvi, V. G., Dodake, S. B. & Joshi, M. S. Interactive effect of bio-organic and inorganic fertilizers on crop growth, quality, productivity and profitability of groundnut (Arachis hypogaea L.) in an Alfisol of Western Ghat of Maharashtra India. *Commun. Soil Sci. Plant Anal.* **56**, 1–19. 10.1080/00103624.2024.2379594 (2025).

[36] Pavan, B. et al. Integrating soil test-based fertilizers and biofertilizers to boost groundnut (Arachis hypogaea) productivity on sandy clay soils in semi-arid tropics. *J. Soil Water Conserv.* **24**, 93–98. 10.5958/2455-7145.2025.00011.6 (2025).

[37] Sahana, G., Murthy, K. N. K., Hanumanthappa, D. C., Anand, M. R. & Raju, B. M. Growth and yield response of groundnut (Arachis hypogaea L.) to different land configurations and planting geometry. *Mysore J. Agric. Sci.* **59**, 331–340 (2025).

[38] Krishnan, K. S. et al. Microbial inoculants—Fostering sustainability in groundnut production. *Sci. Prog.*10.1177/00368504251338943 (2025). 40324969 10.1177/00368504251338943 PMC12059452

[39] Ngmenzuma, T. Y., Oteng-Frimpong, R. & Dakora, F. D. N2 fixation, grain mineral accumulation, and water-use efficiency in 30 field-grown groundnut (Arachis hypogaea L.) genotypes in Mpumalanga, South Africa, measured using 15N and 13C natural abundance techniques. *Front. Agron.* **7**, 1483741. 10.3389/fagro.2025.1483741 (2025).

[40] Mola Bakhsh, M. Z., Ikram, M., Ali, A. & Rehman, H. Heat stress-induced sterility in oilseed crops: A focus on Brassica napus. *Mol. Biol. Rep.* **52**, 652. 10.1007/s11033-025-10694-x (2025). 40581676 10.1007/s11033-025-10694-x

[41] Nourzadeh, N., Rahimi, A. & Dadrasi, A. Modelling the environmental impact of sesame production under different fertilizer and water use regimes. *Sci. Rep.* **15**, 21855. 10.1038/s41598-025-08363-x (2025). 40595259 10.1038/s41598-025-08363-x PMC12217747

[42] Jackson ML (1973) Soil Chemical Analysis. New Delhi: Prentice Hall of India Pvt. Ltd. pp. 498.

[43] Walkley, A. & Black, C. A. An examination to different method for determination soil organic matter and proposal for modification of the chromic acid titration method. *Soil Sci.* **37**, 29–38 (1934).

[44] Olsen SR, Cole CV, Watanabe FS, Dean LA (1954) Estimation of available phosphorus in soil by extraction by sodium bi-carbonate. Cir.USDA939:1–19.

[45] Hanway, J. J. & Heidel, H. Soil analysis methods as used in Iowa state college soil testing laboratory. *Lowa Agric.* **57**, 1–31 (1952).

[46] Williams, C. H. & Steinbergs, A. Soil sulphur fractions as chemical indices of available sulphur in some Australian soils. *Aust. J. Agric. Res.* **10**, 340–352. 10.1071/AR9590340 (1959).

[47] Lindsay, W. L. & Norvell, W. A. Development of DTPA soil test for zinc, iron, manganese and copper. *J. Soil Sci. Soc. Am.* **42**, 421–428 (1978).

[48] Chaudhary, I. A. & Cornfield, A. H. The determination of total sulphur in soil and plant material. *Analyst* **91**, 528–530 (1966).

[49] Parveen K, Banse V, Ledwani L (2016) Green synthesis of nanoparticles: Their advantages and disadvantages. In AIP conference proceedings. AIP Publishing.10.1063/1.4945168.

[50] Fageria, N. K. & Press, C. The use of nutrients in crop plants. *Cereal Res. Commun.* **37**, 149–150. 10.1556/crc.37.2009.1.18 (2009).

[51] Bardsley CE, Lancaster JD (1965) Sulfur. Methods of Soil Analysis: Part 2 Chemical and Microbiological Properties 9: 1102–1116.

[52] ButtersB, C. E. M. A rapid method for the determination of total sulphur in soils and plants. *Analyst* **84**, 239–245 (1959).

[53] Gomez, K. A. & Gomez, A. A. *Statistical procedures for agricultural research* (John wiley& sons, 1984).

[54] Yuan, H. et al. Sulfur nanoparticles improved plant growth and reduced mercury toxicity via mitigating the oxidative stress in Brassica napus L. *Jof. Clean. Prod.* **318**, 128589. 10.1016/j.jclepro.2021.128589 (2021).

[55] Wang, Y. et al. Unraveling how Fe–Mn modified biochar mitigates sulfamonomethoxine in soil water: The activated biodegradation and hydroxyl radicals formation. *J. Hazard. Mater.* **465**, 133490. 10.1016/j.jhazmat.2024.133490 (2024). 38228002 10.1016/j.jhazmat.2024.133490

[56] Gong, H. et al. A dynamic optimization of soil phosphorus status approach could reduce phosphorus fertilizer use by half in China. *Nat. Commun.* **16**, 976. 10.1038/s41467-025-56178-1 (2025). 39856072 10.1038/s41467-025-56178-1 PMC11761064

[57] Yuan, A. et al. Dynamic interplay among soil nutrients, rhizosphere metabolites, and microbes shape drought and heat stress responses in summer maize. *Soil Biol. Biochem.* **191**, 109357. 10.1016/j.soilbio.2024.109357 (2024).

[58] Narayan, O. P., Kumar, P., Yadav, B., Dua, M. & Johri, A. K. Sulfur nutrition and its role in plant growth and development. *Plant Signal Behav.* **18**, 2030082. 10.1080/15592324.2022.2030082 (2023). 35129079 10.1080/15592324.2022.2030082 PMC10730164

[59] Shah, S. H., Islam, S. & Mohammad, F. Sulphur as a dynamic mineral element for plants: A review. *J. Soil Sci. Plant Nutr.* **22**, 2118–2143. 10.1007/s42729-022-00798-9 (2022).

[60] Hasanuzzaman, M. et al. Impact of nano-sulfur on the physiological and molecular responses of mustard (Brassica juncea L.) plants under salt stress. *Plants* **9**, 60 (2020).31906504

[61] Singh VS, Jinger D, Parihar M, Tiwari G, Meena RP, Chitara MK, Jatav SS (2024) Development Prospective and Challenges of Nanotechnology in Sustainable Agriculture. In: Sheraz Mahdi, S., Singh, R., Dhekale, B. (eds) Adapting to Climate Change in Agriculture-Theories and Practices. Springer, Cham. 10.1007/978-3-031-28142-6_10.

[62] Mahajan, R., Singh, N., Parmar, P., Sharma, D. & Jaswal, N. Effect of sulphur and zinc on yield attributes and yield of groundnut (Arachis hypogaea L.). *Leg. Res. Int. J.* **39**, 798–801 (2016).

[63] Rani, R., Agarwal, N., Gaur, V. K. & Singh, B. Influence of integrated nutrient management on growth, yield and quality of mustard (Brassica juncea L.) cv. Pusa Bold. *J. App. Nat. Sci.* **7**, 912–917 (2015).

[64] Ragab, G. A. & Saad-Allah, K. M. Green synthesis of sulfur nanoparticles using Ocimum basilicum leaves and its prospective effect on manganese-stressed Helianthus annuus (L.) seedlings. *Ecotoxic. EnvironSaf.* **191**, 110242. 10.1016/j.ecoenv.2020.110242 (2020). 10.1016/j.ecoenv.2020.110242 32004945

[65] Zhang, D. et al. Remediation of arsenic-contaminated paddy soil: Effects of elemental sulfur and gypsum fertilizer application. *Ecotoxic. Environ. Saf.* **223**, 112606. 10.1016/j.ecoenv.2021.112606 (2021). 10.1016/j.ecoenv.2021.112606 34365211

[66] Yang, X. et al. Sulfur nanoparticles modulate antioxidant defense and methylglyoxal detoxification systems to improve maize growth and tolerance to drought stress. *J. Plant Growth Reg.* **37**, 872–880 (2018).

[67] Rajput, V. D., MinkinaTM, SushkovaSN., MandzhievaSS, FedorenkoAV. & Chaplygin, V. A. Effects of sulfur nanoparticles on growth and nutrient status of mustard plants grown in sandy loam soils. *Environ. Sci. Poll. Res.* **24**, 21145–21154 (2017).

[68] Karimi, E., MehrabiF, GhorbanpourM. & Pakniyat, H. Sulfur nanoparticles improve nutrient uptake and salt tolerance in canola (Brassica napus) under saline conditions. *Comm. Soil Sci. Plant Ana* **49**, 2111–2125 (2018).

[69] Dick WA, Kost D, Chen L (2008) Availability of sulfur to crops from soil and other sources. Sulfur: A missing link between soils, crops, and nutrition 50 59–82.

[70] Bindraban, P. S., Dimkpa, C., Nagarajan, L., Roy, A. & Rabbinge, R. Revisiting fertilisers and fertilisation strategies for improved nutrient uptake by plants. *Bio. Fertof Soils* **51**, 897–911. 10.1007/s00374-015-1039-7 (2015).

[71] Zhang, L. et al. Selenium nanomaterials promoted ferredoxin and iron–sulfur protein synthesis and acetyl CoA carboxylase activity to improve the photosynthesis and fatty-acid synthesis in soybean. *Environ. Sci. Nano* **11**, 2073–2082. 10.1039/D3EN00978E (2024).

[72] Kayata, R., Saharan, K., Kumawat, K. C. & Agrawal, R. D. Effect of foliar application of trace elements and growth regulators on plant biomass and symbiotic efficiency of Lens culinaris M. *Biocat. Agri. Biotech.* **58**, 103169. 10.1016/j.bcab.2024.103169 (2024).

[73] Booali, S., Zoufan, P. & Bavani, M. R. Z. Effect of biofertilizer containing Thiobacillus bacteria along with different levels of chemical sulfur fertilizer on growth response and photochemical efficiency of small radish plants (Raphanus sativus L. var. shushtari) under greenhouse conditions. *Scient. Horticul.* **327**, 112835. 10.1016/j.scienta.2023.112835 (2024).

[74] Abdin MZ, Ahmad A, Khan N, Khan I, Jamal A, Iqbal M (2003) Sulphur interaction with other nutrients. *Sulphur in plants* 359–374.

[75] Das, S., Sengupta, S., Patra, P. K. & Dey, P. Limestone and yellow gypsum can reduce cadmium accumulation in groundnut (Arachis hypogaea): A study from a three-decade old landfill site. *Chemosphere* **353**, 141645 (2024). 38452977 10.1016/j.chemosphere.2024.141645

[76] Falk, K. L., Tokuhisa, J. G. & Gershenzon, J. The effect of sulfur nutrition on plant glucosinolate content: Physiology and molecular mechanisms. *Plant Bio.* **9**, 573–581. 10.1055/s-2007-965431 (2007). 17853357 10.1055/s-2007-965431

[77] Kovar JL, Grant CA (2011) Nutrient cycling in soils: Sulfur. Soil management: Building a stable base for agriculture 10.2136/2011.soilmanagement.c7.

[78] Gangadhara, G. A. & ManjunathaiahHM, S. T. Effect of sulphur on yield, oil content of sunflower and uptake of micronutrients by plants.J. of the Ind. *Soc. Soil Sci.* **38**, 692–695 (1990).

[79] Jankowski KJ, Budzyński, WS, Kijewski Ł, Klasa A (2014) Concentrations of copper, zinc and manganese in the roots, straw and oil cake of white mustard (Sinapis alba L.) and Indian mustard (Brassica juncea (L.) Czern. et Coss.) depending on sulphur fertilization. 60:364–371.

[80] Klikocka, H. & Marks, M. Sulphur and nitrogen fertilization as a potential means of agronomic biofortification to improve the content and uptake of microelements in spring wheat grain DM. *J. Chem.* **18**, 1–12. 10.1155/2018/9326820 (2018).

[81] Babhulkar, P. S., Kar, D., Badole, W. P. & Balpande, S. S. Effect of sulphur and zinc on yield, quality and nutrient uptake by safflower in Vertisol. *J. Ind. Soc. Soil Sci.* **48**, 541–543 (2000).

[82] Malewar GU, Ismail S (1997) Sulphur in balanced fertilization in western India. In Proceedings of the TSI/FAI/IFA Symposium on Sulphur in Balanced Fertilization, New Delhi 14, pp. 336-404.

[83] Balanagoudar, S. R. & Satyanarayana, T. Depth distribution of different forms of sulphur in Vertisols and Alfisols. *J. Ind. Soc. Soil Sci.* **38**, 634–640 (1990).

[84] Gowrisankar, D. & Shukla, L. M. Sulphur forms and their relationship with soil properties in Inceptisols of Delhi. *J. Indian Soc. Soil Sci.* **47**, 437–442 (1999).

[85] Jat, J. R. & Yadav, B. L. Different forms of sulphur and their relationship with properties of Entisols of Jaipur district (Rajasthan) under mustard cultivation. *J. Ind. Soc. Soil. Sci.* **54**, 208–212 (2006).

[86] Basumatary, A., Talukdar, M. C. & Ramchiary, S. Sulphur forms and their relationship with soil properties in rapeseed growing soils of Upper Assam. *Int. J. Trop. Agri.* **26**, 69–72 (2008).

[87] Yadav, S. L. et al. Impact of sulphur nanoparticles on growth and biological yield of groundnut-mustard crop sequence under sandy loam soils of central Gujarat. *Int. J. Plant Soil Sci.* **35**, 1105–1112 (2023).

[88] Shivay, Y. S. & Prasad, R. Zinc-coated urea improves productivity and quality of basmati rice (*Oryza sativa* L.) under zinc stress condition. *J. Plant Nutri.* **35**, 928–951. 10.1080/01904167.2012.663444 (2012).

[89] Khater AM (1981) A study of sulphur and petroleum by products as efficient materials affecting the availability of certain nutrients in soil, DissertationShams Univ., 1981, Accessed on 12 May, 2024.

[90] Mostafa MA, El-Gala AM, Wassif MM, El-Maghraby SE (1992) Distribution of some micronutrients through a calcareous soil column under sulphur and saline water application. Proceedings Middle East Sulphur Symposium 12–16 February, Cairo, Egypt, 1990, pp 263–276.

[91] Prasad, R. & Shivay, Y. S. Sulphur fertilization and food quality-A review. *Ind J of Agro* **62**, 1–7 (2017).

[92] Ryan, J., Miyamoto, S. & Stroehlein, J. L. Solubility of manganese, iron, and zinc as affected by application of sulfuric acid to calcareous soils. *Plant Soil* **40**, 421–427. 10.1007/BF00011527 (1974).

[93] Singh, R., Dwivedi, B. S. & NayakAK, S. V. K. Effect of sulphur and zinc application on soil available nutrients and yield of groundnut (Arachis hypogaea) and mustard (Brassica juncea) cropping sequence. *Ind. J. Argon.* **64**, 437–442 (2019).

[94] Yousry, M., El-Leboudi, A. & Khater, A. Effect of sulphur and petroleum by-products on soil characteristics. 2.-Availability of some nutrients in a calcareous soil [Egypt]. *Egyp J. Soil Sci.* **24**, 195–200 (1984).

