# Supplementary material for: Nanotechnology strategies for enhancing productivity, sulphur use efficiency and soil fertility in groundnut-mustard cropping systems
Source: Sci Rep. 2026 Jan 6;16:3170. doi: 10.1038/s41598-025-33174-5 (PMC12830652; doi:10.1038/s41598-025-33174-5)
Supplement: Supplementary file 1 — Supplementary Material 1 [file 41598_2025_33174_MOESM1_ESM.docx]

**Fig. S1: Effect of SNPs and elemental sulphur on sulphur content in different plant parts of groundnut**

**Fig. S2: Effect of SNPs and elemental sulphur on sulphur content in different plant parts of mustard**
